# XPC Deficiency Activate Cisplatin‐Mediated Autophagy in Bladder Cancer by Limiting Novel PHRF1‐Mediated Ubiquitination of the p53 Protein

**DOI:** 10.1002/advs.202517563

**Published:** 2025-11-11

**Authors:** Baixiong Zhao, Yaqin Huang, Jiazhong Shi, Xiaozhou Zhou, Johan Bourghardt Fagman, Liwei Wang, Sha Liu, Wuxing Wang, Yuting Liu, Zhiwen Chen, Jin Yang

**Affiliations:** ^1^ Department of Urology The First Affiliated Hospital (Southwest Hospital) of Army Medical University Chongqing 400038 China; ^2^ Department of Cell Biology Army Medical University Chongqing 400038 China; ^3^ Department of Surgery Institute of Clinical Sciences Sahlgrenska Academy University of Gothenburg Gothenburg 41345 Sweden; ^4^ Department of Surgery Sahlgrenska University Hospital Gothenburg 41345 Sweden

**Keywords:** autophagy, bladder cancer, drug resistance, p53, PHRF1, XPC

## Abstract

Muscle‐invasive bladder cancers (MIBC) are biologically heterogeneous and have widely variable conventional chemotherapy responses and clinical outcomes. This study demonstrates that XPC deficiency in bladder cancer cells can promote autophagy in response to the cisplatin‐mediated DNA damage response (DDR). This process is closely related to both the overexpression of KDM4A and the downregulation of PHRF1 induced by the overactivation of ATM phosphate. The overaccumulation of KDM4A can suppress PHRF1 expression and result in significant nuclear accumulation of the p53 protein. Notably, this study defines a new mechanism by which PHRF1 regulates p53 posttranslationally through the ubiquitin‐proteasome system. In XPC low expression cells, PHRF1 performs a more critical E3 ubiquitin ligase function than MDM2. Especially under conditions of cisplatin‐mediated DNA damage where MDM2 function is impaired, PHRF1 retains its functionality. In a mouse xenograft model, combining a KDM4 inhibitor with cisplatin results in superior antitumor effects compared with cisplatin alone. These findings provide new insights into the phenotypic plasticity of bladder cancer under drug resistance and highlight the potential of KDM4A inhibition and preservation of PHRF1 function in overcoming cisplatin resistance. Therefore, KDM4A or PHRF1 may be potential novel targets for the treatment of bladder cancer.

## Graphical abstract

This study reveals that XPC regulates autophagy signaling beyond its noncanonical DNA repair role, uncovering the XPC‐p‐ATM‐KDM4A‐PHRF1 axis as a novel mechanism for autophagy modulation under DNA damage stress. It identifies PHRF1 as a novel p53 ubiquitin ligase. The findings suggest that targeting PHRF1 function and inhibiting KDM4A may represent a potential strategy to overcome cisplatin resistance.

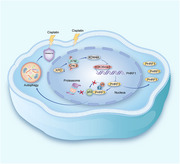

## 1 Introduction

Drug resistance refers to the process by which cancer cells develop resistance to systemic chemotherapy, targeted therapy, immunotherapy, or other antitumor drugs.^[^ ^1^ ^]^ The development of drug resistance in cancer is a complex, multifactorial process. It occurs through multiple mechanisms, including increased drug efflux, decreased drug uptake, target mutations, alterations in signaling pathways, phenotype switching, and defects in or activation of apoptosis and autophagy.^[^ ^2^, ^3^ ^]^

Genomic DNA in cells is continuously damaged by both external and internal (endogenous) factors. DNA repair is crucial for maintaining genetic stability and fidelity within cells.^[^ ^4^ ^]^ Nucleotide excision repair (NER) is one of the major pathways among the various DNA repair mechanisms.^[^ ^5^ ^]^ XPC is the earliest acting DNA damage recognition protein involved in NER, and it directly facilitates DNA repair. Recent studies have revealed that XPC also plays a significant signaling role in biological processes such as apoptosis and cell cycle dysregulation in response to DNA damage.^[^ ^6^, ^7^, ^8^ ^]^ Consequently, defects or mutations in XPC can affect multiple biological processes following DNA damage. Macroautophagy (referred to as autophagy) is a conserved metabolic process in which autophagosomes fuse with lysosomes to degrade damaged proteins and organelles, maintaining cellular homeostasis.^[^ ^9^ ^]^ Nutrient deprivation, metabolic abnormalities, oxidative stress, or pathological stimuli induce cellular stress to promote autophagic flux, helping to preserve cellular function as an adaptive response.^[^ ^10^ ^]^ The interaction between the DDR and autophagy dictates cellular fate, ultimately influencing biological behavior. Research indicates that autophagy has a broad role in tumorigenesis, invasion, metastasis, chemoresistance, and stem cell maintenance.^[^ ^11^, ^12^, ^13^ ^]^ However, the relationship between XPC and autophagy is still unclear.

Variations in or loss of the XPC gene are linked to the development of solid tumors and hematologic malignancies in humans.^[^ ^14^, ^15^ ^]^ Previous molecular epidemiology studies, along with our research, have shown that loss of XPC gene expression is strongly associated with the onset and progression of bladder cancer, forming a genetic basis for the development and heterogeneity of this disease.^[^ ^16^ ^]^ XPC loss frequently occurs in bladder cancer and significantly affects its prognosis. Furthermore, most bladder cancer patients relapse after chemotherapy, resulting in faster progression and increased malignancy.^[^ ^7^, ^16^ ^]^ In this study, we identified strong correlations among XPC deficiency in MIBC, cellular autophagy, and drug resistance. These findings led us to explore how XPC expression regulates autophagy and drug resistance in bladder cancer cells. During this investigation, we identified a novel E3 ubiquitin ligase, PHRF1, which targets p53 as its substrate.

## 2 Results

### 2.1 MIBC Patients with XPC Deficiency Exhibit Poorer Neoadjuvant Chemotherapy (NAC) Responsiveness based on Cisplatin and Poorer Survival Outcomes

**Figure 1:**
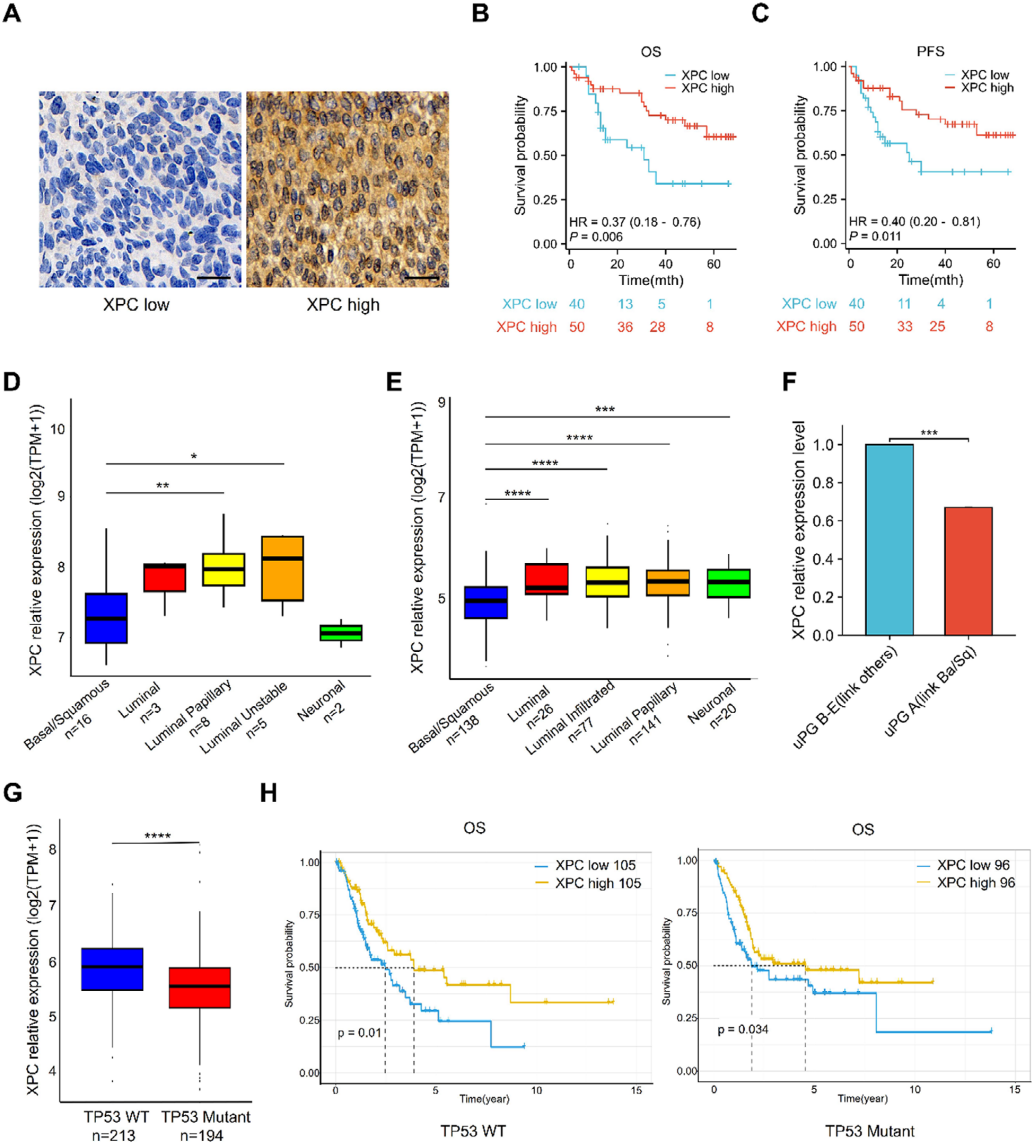
MIBC patients with XPC deficiency exhibit poorer neoadjuvant chemotherapy responsiveness based on cisplatin and poorer survival outcomes. Immunohistochemical staining and analysis of XPC were performed on tissue microarrays from 90 MIBC patients. Utilizing XPC expression levels as a variable, statistical analyses were conducted based on OS and PFS data obtained from clinical follow‐up records. Statistical analysis of XPC expression was performed based on the TCGA database and recently published multi‐omics databases of bladder cancer. A) Representative images of immunohistochemical staining of XPC. Scale bar: 30 µm. B) OS analysis of 90 patients with MIBC grouped according to XPC expression levels. C) PFS analysis of 90 patients with MIBC grouped according to XPC expression levels. D) Comparison of XPC mRNA expression levels in patients with different molecular subtypes of bladder cancer in Groeneveld's database (PMID: 37 380 559). E) Comparison of XPC mRNA expression levels in patients with different molecular subtypes of bladder cancer in the TCGA database. F) The comparison of XPC protein expression levels between the uPG‐A subtype and other subtypes in the proteomic database of untreated bladder cancer published by Groeneveld et al (PMID: 37 380 559). G) Differential analysis of XPC expression in bladder cancer patients from the TCGA database, stratified by TP53 mutation status. H) OS curves of bladder cancer patients with different TP53 statuses from the TCGA database stratified by the median expression level of XPC. In each panel, Bars represent group means. *p < 0.05, **p < 0.01, ***p < 0.001, ****p < 0.0001. Abbreviations: OS overall survival, PFS progression‐free survival, MIBC muscle‐invasive bladder cancer, NAC neoadjuvant chemotherapy, TCGA The Cancer Genome Atlas, WT wild type.

Our previous research revealed that XPC loss frequently occurs in patients with bladder cancer and significantly affects their survival outcomes.^[^ ^7^ ^]^ We collected tumor tissue samples and clinical data from 90 MIBC patients who had not undergone radiotherapy or chemotherapy. The median age of this cohort was 65 years, with male patients accounting for 88.9% (**Table** **1**). Immunohistochemistry revealed that 40 (44.44%) of these bladder cancer tissues exhibited loss of XPC expression (**Figure** **1**A). The results revealed that patients with low XPC expression had significantly worse overall survival (OS) and progression‐free survival (PFS) than those with high XPC expression (Figure 1B,C).

**Table 1:** Clinical characteristics of MIBC tumor tissue cohorts.

Clinical variable	XPC low, *N* = 40	XPC high, *N* = 50
Median age, years (range)	65 (44–85)	65 (38–77)
Sex, *n* (%)		
Male	36 (40.00%)	44 (48.89%)
Female	4 (4.44%)	6 (6.67%)
Average height, cm (range)	163.05 (150–175)	163.76 (150–174)
Average weight, kg (range)	63.08 (44–94)	59.53 (45–80)
Average BMI, kg m^−2^(range)	23.67(16.90–37.18)	22.11(17.21–27.34)
Smoking status, *n* (%)		
No	14(15.56%)	15(16.67%)
Yes	26(28.89%)	35(38.88%)
ypT‐stage, *n* (%)		
ypTa	‐	‐
ypT1	‐	‐
ypT2	27(30.00%)	38(42.22%)
ypT3	8(8.89%)	6(6.67%)
ypT4	5(5.55%)	6(6.67%)
ypN‐stage, *n* (%)		
ypN0	26(28.89%)	43(47.78%)
ypN1	3(3.33%)	2(2.22%)
ypN2	10(11.11%)	5(5.56%)
Not available	1(1.11%)	
p53 status, *n* (%)		
p53 wt	19(21.11%)	23(25.56%)
p53 mutation	21(23.33%)	27(30.00%)

Recently published multi‐omics data from different regions were also incorporated into the study. Groeneveld et al. investigated 300 MIBC patients treated with NAC (both regimens cisplatin‐based) and found that the Basal/Squamous subtype exhibited the poorest therapeutic response, significantly lower than other subtypes.^[^ ^17^ ^]^ Contreras‐Sanz et al. conducted a proteomic comparison of 55 platinum‐based NAC‐treated MIBC patients pre‐ and post‐therapy, revealing that the CC3 subtype (linked to Basal/Squamous subtype) showed significantly reduced platinum sensitivity compared to other subtypes.^[^ ^18^ ^]^ Additional analysis of a recently released multiomics raw dataset^[^ ^19^ ^]^ from untreated bladder cancer patients revealed that the Basal/Squamous subtype exhibited significantly lower XPC mRNA expression compared to the Luminal Papillary and Luminal Unstable subtypes (Figure 1D). The lack of significant differences between the Basal/Squamous and Luminal /Neuronal subtypes may be attributed to limited sample sizes (3 and 2 cases for Luminal and Neuronal subtypes, respectively). TCGA database analysis further demonstrated that XPC expression was markedly lower in Basal/Squamous‐subtype bladder cancer patients than in other subtypes (Figure 1E). Additional analysis of the proteomics raw data^[^ ^19^ ^]^ from this dataset revealed that XPC expression was significantly downregulated in the uPG‐A subtype (linked to Basal/Squamous subtype) (logFC = −0.57, *p* = 0.0202, AUC = 0.86) (Figure 1F). TP53 mutation is one of the important clinical markers for poor prognosis in MIBC patients. We analyzed XPC expression levels in TP53 wild‐type and mutant MIBC patients from the TCGA database, and the results showed that XPC expression was significantly lower in TP53 mutant cases compared to wild‐type (Figure 1G). Subsequently, we analyzed the impact of XPC expression on OS in TP53 wild‐type and mutant MIBC patients from the TCGA database. The results demonstrated that low XPC expression was associated with poor prognosis, regardless of TP53 mutation status (Figure 1H). Collectively, these findings suggest that low XPC expression is a strong predictor of a poor bladder cancer prognosis and a poor cisplatin sensitivity; this trend can be observed across different populations worldwide.

### 2.2 XPC‐Knockdown Bladder Cancer Cell Lines Exhibit Cisplatin Resistance

**Figure 2:**
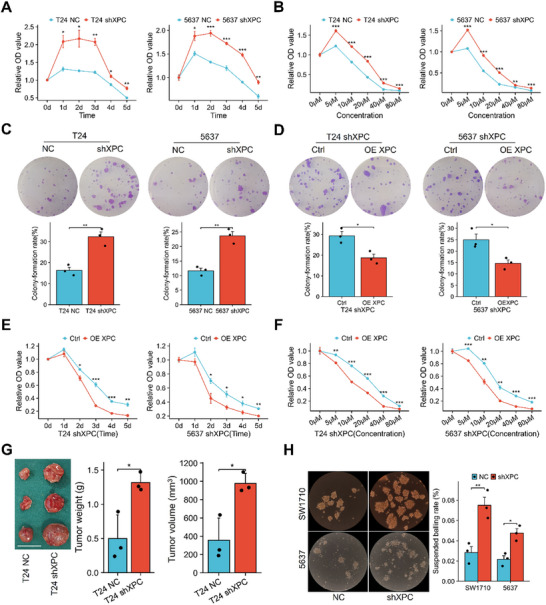
XPC‐knockdown bladder cancer cell lines exhibit cisplatin resistance. In vitro experiments were conducted by establishing T24 and 5637 cell lines with stable XPC knockdown. The cells' sensitivity to cisplatin was assessed at different time points under treatment with 5 µm cisplatin. Subsequently, with cisplatin exposure time fixed at 3 d (removed after 4 h of treatment), cellular drug sensitivity was evaluated under varying concentrations of cisplatin. In the clonogenic assay, cisplatin was used at concentrations of 20 µm for T24 cells or 10 µm for 5637 cells. After restoring XPC expression using a eukaryotic XPC plasmid, all the aforementioned experiments were repeated. Details of the suspension sphere formation experiment and in vivo experiments are described in the Methods section. A) CCK‐8 assay results for T24 and 5637 cells after 1–5 d of stimulation with 5 µm cisplatin (*n* = 3). B) CCK‐8 assay results for T24 and 5637 cells after 3 days of stimulation with different concentrations of cisplatin (*n* = 3). C) Colony formation assay results for T24 cells stimulated with 20 µm cisplatin and 5637 cells stimulated with 10 µm cisplatin (*n* = 3). D) Colony formation assay results for XPC‐reconstituted T24 shXPC and 5637 shXPC cells stimulated with 20 µm cisplatin (T24) and 10 µm cisplatin (5637) (*n* = 3). E) CCK‐8 assay results for XPC‐reconstituted T24 shXPC and 5637 shXPC cells after 1–5 d of stimulation with 20 µm cisplatin (T24) or 10 µm cisplatin (5637) (*n* = 3). F) CCK‐8 assay results for XPC‐reconstituted T24 shXPC and 5637 shXPC cells after 3 d of stimulation with different concentrations of cisplatin (removed after 4 h of treatment) (*n* = 3). G) Xenograft tumors in nude mice after 4 weeks of cisplatin treatment (5 mg/kg body weight, once per week, via intraperitoneal injection) (*n* = 3). Scale bar: 1 cm. H) Spheres formed by SW1710 and 5637 cells after 2 weeks of suspension culture (*n* = 3). For each animal or independent cell culture experiment, measurements were repeated three times under the same experimental conditions. In each panel, dots represent individual measurements from animals or independent cell culture experiments. Bars represent group means, and error bars indicate ± standard deviation (SD). *p < 0.05, **p < 0.01, ***p < 0.001. Abbreviations: CCK‐8 Cell Counting Kit‐8, CCLE Cancer Cell Line Encyclopedia, cis cisplatin.

We investigated the basal XPC expression levels across multiple human bladder cancer cell lines through the CCLE database (Figure S1, Supporting Information). Based on tumor epidemiology data demonstrating that XPC deficiency is a frequent event in bladder cancer and that XPC deficiency correlates with tumor malignancy, we selected T24 (moderate XPC expression) and 5637 (low XPC expression) cells as research models. Basal subtype cell lines are considered the preferred models for studying cisplatin resistance in bladder cancer due to their high molecular similarity to clinically resistant patients. Both T24 and 5637 cell lines are classified as basal subtype, although the CCLE database also categorizes T24 cells as nonbasal, nonluminal, potentially due to culturing or profiling artifacts.^[^ ^20^ ^]^ We used shRNA to knock down XPC expression in T24 and 5637 cells (based on previous studies from our research group^[^ ^8^ ^]^) and used cisplatin, a common clinical chemotherapy drug, as a stimulus. First, the CCK‐8 assays results revealed that XPC‐knockdown cells presented significant cisplatin resistance at multiple time points and different concentrations of cisplatin (**Figure** **2**A,B). Furthermore, the colony formation assay results revealed a significant increase in the colony formation rate of XPC‐knockdown cells (Figure 2C). We introduced a eukaryotic XPC plasmid into XPC‐knockdown cells to restore XPC expression. The colony formation assay revealed significantly reduced colony formation rates (Figure 2D). Moreover, the CCK‐8 assay results indicated that after XPC expression was restored, cells exhibited increased drug sensitivity(Figure 2E,F). Furthermore, in vivo experiments results revealed that the final tumor mass derived from XPC‐knockdown cells was significantly greater than that derived from control cells, indicating a marked decrease in cisplatin sensitivity (Figure 2G). Many studies have revealed that cancer stem cell proportions are closely associated with chemotherapy resistance.^[^ ^21^, ^22^ ^]^ We used the classical sphere formation assay to evaluate the sphere‐forming ability of cells after XPC knockdown. As T24 cells could not form spheres in culture system, we included SW1710 cells for evaluation. The results revealed a significant increase in the number of spheres formed after XPC knockdown (Figure 2H). In conclusion, these findings indicate that low XPC expression drives chemotherapy resistance in bladder cancer.

### 2.3 Loss of XPC in Bladder Epithelial Tissues of XPC Knockout Mice is Linked to Autophagy Activation

**Figure 3:**
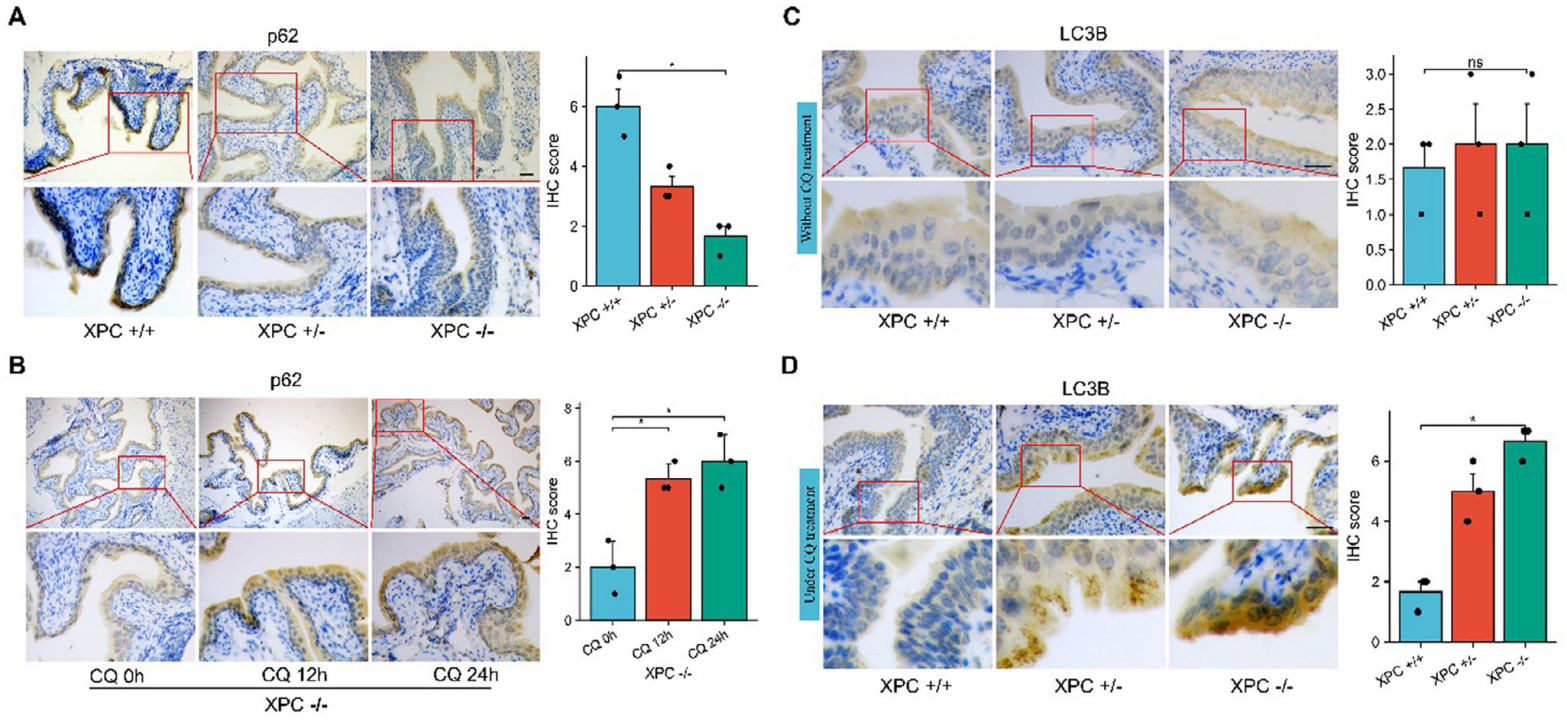
Loss of XPC in bladder epithelial tissues of XPC knockout mice is linked to autophagy activation. Immunohistochemical staining was performed on normal bladder epithelial tissues obtained from XPC knockout mice. The CQ treatment mice were administered drugs via intraperitoneal injection. The drug concentration was 80 mg/kg, with a single intraperitoneal injection. The mice were euthanized 12 or 24 h later, and samples were collected. A) Immunohistochemical analysis of p62 in the bladder transitional epithelium of XPC knockout mice (*n* = 3). Scale bar: 50 µm. B) Immunohistochemical analysis of p62 in the bladder transitional epithelium of XPC knockout mice after 12 or 24 h of CQ treatment (*n* = 3). Scale bar: 50 µm. C) Immunohistochemical analysis of LC3B in the bladder transitional epithelium of XPC knockout mice (*n* = 3). Scale bar: 50 µm. D) Immunohistochemical analysis of LC3B in the bladder transitional epithelium of XPC knockout mice after 24 h of CQ treatment (*n* = 3). Scale bar: 50 µm. For each animal experiment, measurements were repeated three times under the same experimental conditions. In each panel, dots represent individual measurements from animal experiments. Bars represent group means, and error bars indicate ± standard deviation (SD). *p < 0.05, ns, not significant. Abbreviations: CQ chloroquine.

Growing evidence indicates that autophagy plays a broad role in tumorigenesis, invasion, metastasis, chemoresistance, and stem cell maintenance.^[^ ^23^, ^24^, ^25^ ^]^ We examined the expression of the autophagy markers in the normal bladder transitional epithelium of XPC knockout mice. Under basal conditions, the expression of p62 protein in XPC‐/‐ mice was significantly decreased compared to wild‐type mice (**Figure** **3**A). To exclude the potential influence of impaired proteasomal degradation pathway, XPC‐/‐ mice were treated with chloroquine (CQ) via intraperitoneal injection. A gradual increase in p62 protein was observed at 12‐ and 24‐h post‐treatment, indicating unimpaired autophagic flux in XPC‐/‐ mice (Figure 3B). In the basal state, there was no significant change in LC3B‐positive puncta in XPC‐/‐ mice (Figure 3C). However, the results demonstrated a significant accumulation of the LC3B‐positive puncta in XPC‐/‐ mice under the treatment of CQ, compared to XPC+/+ mice (Figure 3D). It is noteworthy that in the immunohistochemical results of LC3B, positive signals exhibited a punctate pattern exclusively localized in the cytoplasm, which is consistent with the characteristic distribution of autophagosomes in cells. This finding from animal models strongly suggest a close relationship between XPC and autophagy.

### 2.4 XPC Knockdown in Bladder Cancer Cells induces Cisplatin Resistance through Autophagy Activation

**Figure 4:**
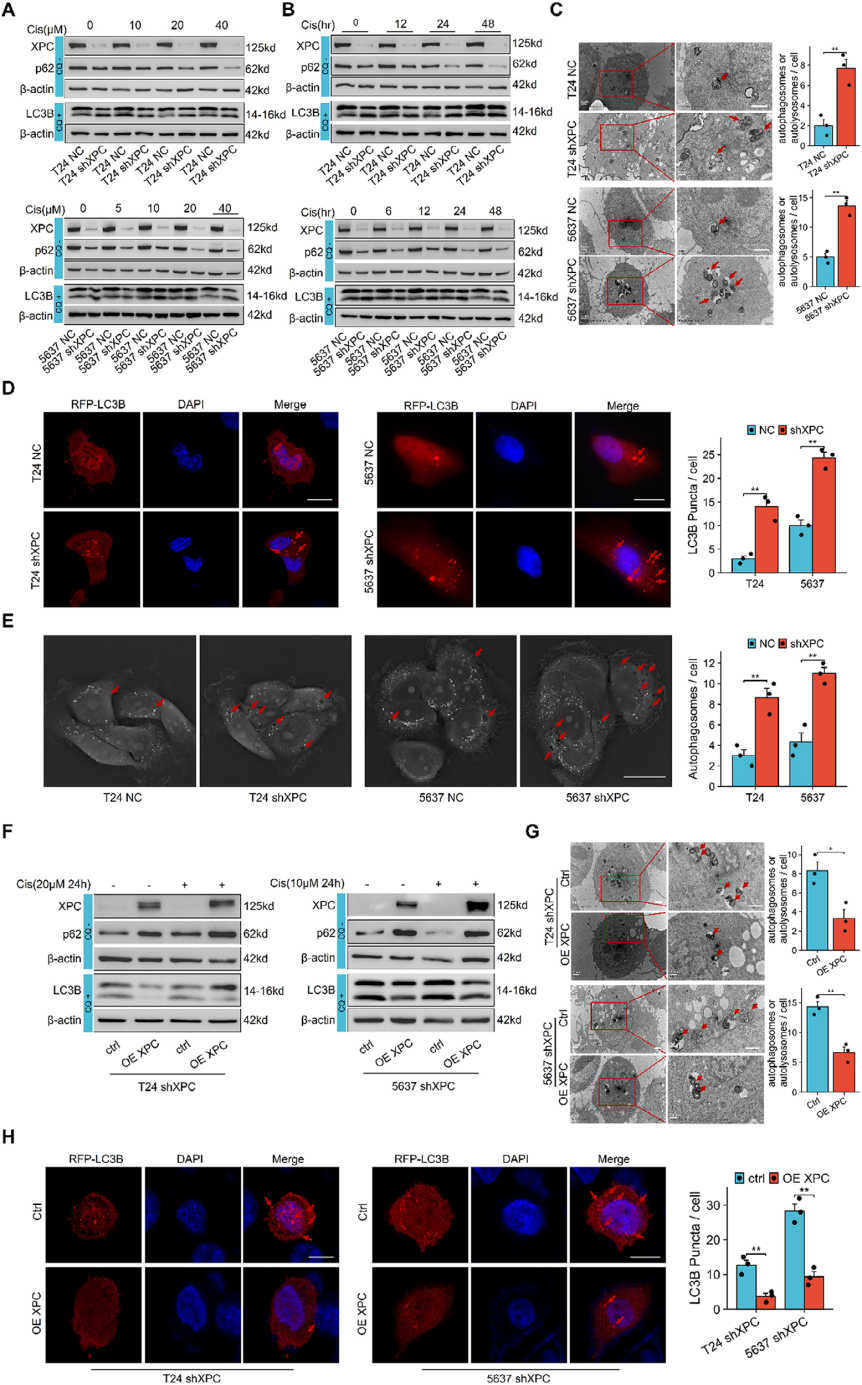
XPC knockdown in bladder cancer cells significantly promotes autophagy activation. In vitro, T24 and 5637 cells were initially treated with varying concentrations of cisplatin (removed after 4 h of treatment). After 24 h, total cellular proteins were harvested for Western blot analysis. The cells were then treated with specific cisplatin concentrations (20 µm for T24 cells and 10 µm for 5637 cells) (removed after 4 h of treatment), and total proteins were collected at different time points for autophagy‐related protein detection. For TEM experiments, cells were treated with the designated cisplatin concentrations (20 µm for T24 cells and 10 µm for 5637 cells) for 24 h before fixation. Similarly, for RFP‐LC3B plasmid transfection experiments, cells were treated with the same cisplatin concentrations (20 µm for T24 cells and 10 µm for 5637 cells) for 24 h prior to fluorescence microscopy observation. CQ was rigorously applied as a control factor (4 h, 10 µm) in all these experiments. Finally, XPC eukaryotic plasmids were transfected into stably XPC‐knockdown cells to restore XPC expression, and the aforementioned experiments (Western blot, TEM, and RFP‐LC3B fluorescence microscopy) were repeated under the same CQ control conditions as previously described. A) Western blot analysis of LC3B and p62 protein levels in T24 and 5637 cells treated with various concentrations of cisplatin for 24 h. When detecting LC3B, 10 µm CQ treatment for 4 h was used as the control condition. B) Western blot analysis of LC3B and p62 protein levels in T24 and 5637 cells treated with cisplatin (20 µm for T24 cells, 10 µm for 5637 cells) for 0–48 h. When detecting LC3B, 10 µm CQ treatment for 4 h was used as the control condition. C) TEM images showing autophagosomes or autolysosomes in T24 and 5637 cells (*n* = 3), 10 µm CQ treatment for 4 h was used as the control condition. Scale bar: 1 µm. D) Fluorescence microscopy images of RFP‐LC3B in T24 and 5637 cells (*n* = 3), 10 µm CQ treatment for 4 h was used as the control condition. Scale bar: 10 µm. E) 3D holographic imaging of autophagosomes or autolysosomes in T24 and 5637 cells (*n* = 3). Scale bar: 20 µm. F) Western blot analysis of LC3B and p62 protein levels in XPC‐reconstituted T24 and 5637 shXPC cells cultured for 1 d in media with or without cisplatin (20 µm for T24, 10 µm for 5637). When detecting LC3B, 10 µm CQ treatment for 4 h was used as the control condition. G) TEM images showing autophagosomes or autolysosomes in XPC‐reconstituted T24 and 5637 shXPC cells (*n* = 3), 10 µm CQ treatment for 4 h was used as the control condition. Scale bar: 1 µm. H) Fluorescence microscopy images of RFP‐LC3B in XPC‐reconstituted T24 and 5637 shXPC cells (*n* = 3), 10 µm CQ treatment for 4 h was used as the control condition. Scale bar: 10 µm. For each independent cell culture experiment, measurements were repeated three times under the same experimental conditions. In each panel, dots represent individual measurements from independent cell culture experiments. Bars represent group means, and error bars indicate ± standard deviation (SD). *p < 0.05, **p < 0.01. Abbreviations: cis cisplatin, TEM Transmission electron microscopy, CQ chloroquine.

To investigate the regulatory relationship between XPC and autophagy, cells were treated with different concentrations of cisplatin for 24 h (removed after 4 h of treatment). The Western blot results showed that XPC knockdown decreased p62 levels, and under CQ intervention, the accumulated amplitude of the LC3B‐II/LC3B‐I ratio after XPC knockdown compared to the basal state is significantly increased (**Figure** **4**A). Total protein was collected at various time points for Western blot analysis. The results confirmed that XPC knockdown significantly activated autophagy (Figure 4B). Transmission electron microscopy (TEM) results further confirmed that XPC knockdown significantly increased the accumulation of autophagosomes or autolysosomes after CQ intervention (Figure 4C). T24 and 5637 cells were transfected with RFP‐labelled LC3B plasmids, and fluorescence microscopy revealed a marked increase in the accumulation degree of LC3B‐positive puncta in XPC‐knockdown cells under CQ treatment (Figure 4D). Repeated observations under baseline conditions (without CQ), including LC3B expression, the number of autophagosomes/autolysosomes under TEM, and RFP‐LC3B‐positive puncta, consistently showed no significant differences (Figure S2, Supporting Information). Thus, subsequent experiments omitted the non‐CQ baseline control group. Additionally, 3D holographic imaging captured autophagosomes or autolysosomes formation in T24 and 5637 cells, supporting these findings (Figure 4E). We also assessed the impact of XPC knockdown on apoptosis and detected no changes in the expression of the apoptosis‐related protein Caspase‐3 in this model (Figure S3, Supporting Information). We reintroduced XPC into T24 shXPC and 5637 shXPC cells via a eukaryotic plasmid and reassessed autophagy‐related markers. The results revealed that XPC reintroduction significantly increased p62 expression and decreased the accumulation of the LC3B‐II/LC3B‐I ratio and reduced the accumulation of RFP‐LC3B positive puncta and autophagosomes or autolysosomes (Figure 4F–H).

To investigate the role of autophagy in bladder cancer chemotherapy resistance, we used CQ, an autophagy inhibitor, combined with cisplatin. The results of the CCK‐8 assay revealed that the combination of cisplatin and CQ had a stronger cytotoxic effect than did cisplatin alone (Figure S4A, Supporting Information). As expected, the colony formation assay supported these results (Figure S4B, Supporting Information). In a nude mouse tumor xenograft model, the results revealed that the combination of cisplatin and CQ resulting in the smallest tumor size (Figure S4C, Supporting Information). Then, we used CQ to block autophagy. The results of the CCK‐8 assay and colony formation assay revealed that XPC knockdown did not affect the sensitivity of bladder cancer cells to cisplatin (Figure S4D,E, Supporting Information). In conclusion, these experiments demonstrate that abnormal autophagy activation is a key factor in the resistance to chemotherapy induced by XPC deficiency in bladder cancer.

### 2.5 XPC Knockdown in Bladder Cancer Cells Activates Autophagy by Increasing p‐ATM Expression

**Figure 5:**
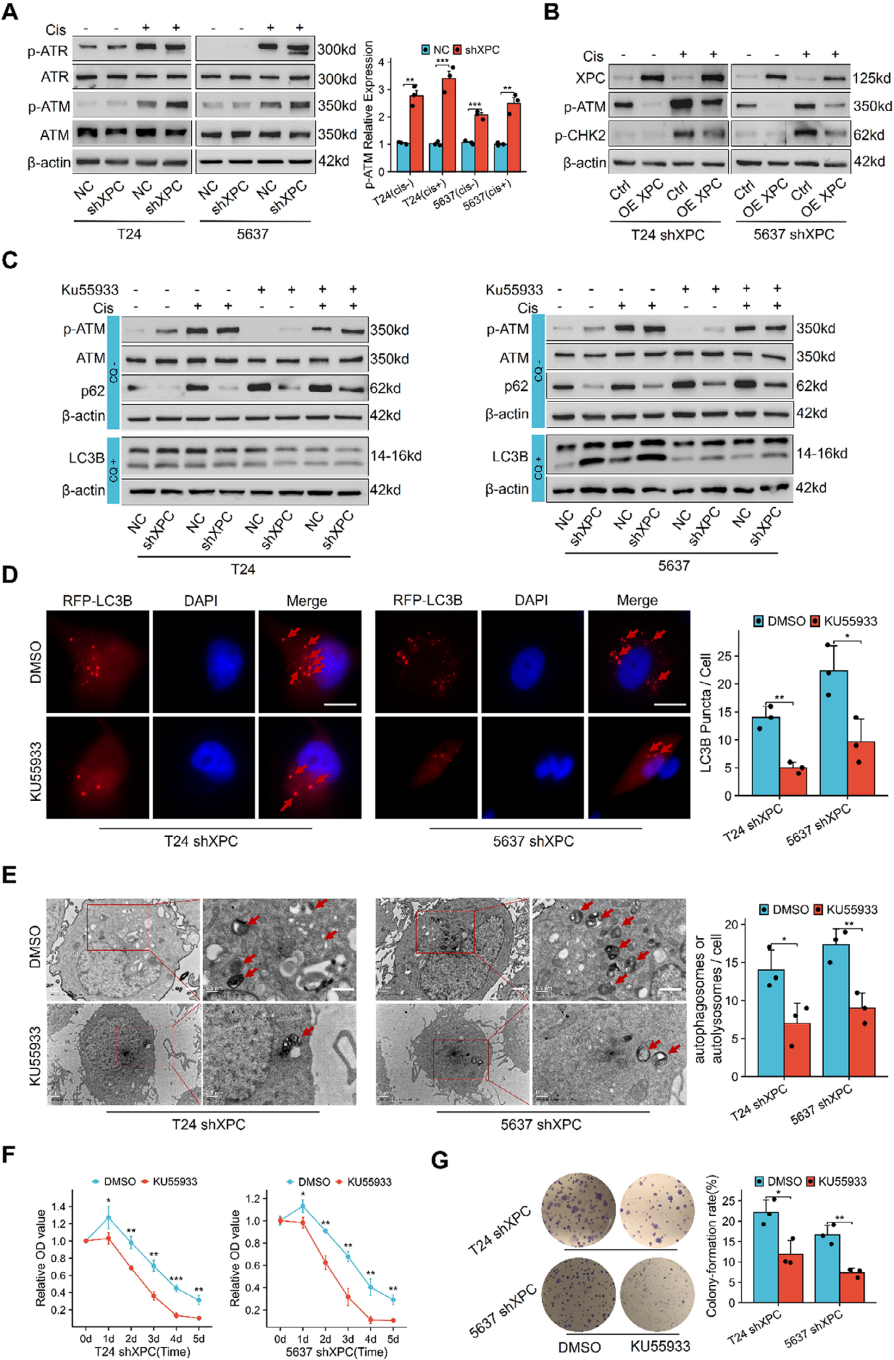
XPC knockdown in bladder cancer cells induces autophagy by increasing p‐ATM expression. In vitro, XPC knockdown and rescue experiments demonstrated the regulatory role of XPC on p‐ATM. In the p‐ATM rescue experiments, cisplatin was maintained at concentrations of 20 µm for T24 cells and 10 µm for 5637 cells. After 24 h (with 4 h of cisplatin treatment), total cellular proteins were collected. In different cells, KU55933 was used at a concentration of 0.5 µm for 24 h. For Western blot analysis of LC3B, TEM examination, and RFP‐LC3B plasmid transfection experiments, CQ was employed as a control condition following the same treatment protocols as previously described. During cisplatin sensitivity testing, cisplatin was maintained at concentrations of 20 µm for T24 cells and 10 µm for 5637 cells, while KU55933 was consistently used at 0.5 µm across all cell lines. A) Western blot analysis of ATM and ATR kinase proteins in T24 and 5637 cells (*n* = 3). B) Western blot analysis of p‐ATM and p‐CHK2 in T24 and 5637 shXPC cells after XPC restoration via XPC eukaryotic plasmid overexpression. C) Western blot analysis of p‐ATM, LC3B, and p62 protein levels in T24 and 5637 cells after 24 h of treatment with KU55933 (0.5 µm) and cisplatin (20 µm for T24 cells, 10 µm for 5637 cells). When detecting LC3B, 10 µm CQ treatment for 4 h was used as the control condition. D) Fluorescence microscopy of RFP‐LC3B puncta in T24 and 5637 shXPC cells after KU55933 treatment (0.5 µm, 24 h) (*n* = 3), 10 µm CQ treatment for 4 h was used as the control condition. Scale bar: 10 µm. E) TEM of T24 and 5637 shXPC cells after KU55933 treatment (0.5 µm, 24 h) (*n* = 3), 10 µm CQ treatment for 4 h was used as the control condition. Scale bar: 1 µm. F) CCK‐8 assay results for KU55933‐treated (0.5 µm) T24 shXPC and 5637 shXPC cells after 1–5 d of stimulation with 20 µm cisplatin (T24) or 10 µm cisplatin (5637) (*n* = 3). G) Colony formation assay results for KU55933‐treated (0.5 µm) T24 shXPC and 5637 shXPC cells stimulated with 20 µm cisplatin (T24) and 10 µm cisplatin (5637) (*n* = 3). For each independent cell culture experiment, measurements were repeated three times under the same experimental conditions. In each panel, dots represent individual measurements from independent cell culture experiments. Bars represent group means, and error bars indicate ± standard deviation (SD). *p < 0.05, **p < 0.01, ***p < 0.001. Abbreviations: cis cisplatin, TEM Transmission electron microscopy, CQ chloroquine, DDR DNA damage response.

In response to DNA damage, ATM/ATR kinase‐dependent signaling pathways are activated to mitigate harmful effects.^[^ ^26^, ^27^ ^]^ Previous studies reported that in budding yeast treated with the alkylating agent ethyl methanesulfonate (a DNA crosslinking agent), ATM/ATR activation upregulates the expression of autophagy‐related genes.^[^ ^28^ ^]^ This discovery suggests that genome toxicity‐induced DDR may activate autophagy through mechanisms distinct from classical pathways. Western blot analysis revealed that after XPC knockdown, p‐ATM level was significantly increased, and these levels were further increased with cisplatin treatment (**Figure** **5**A). We then restored XPC expression. The results revealed that the levels of p‐ATM and its substrate p‐CHK2 were markedly reduced (Figure 5B). We treated T24 and 5637 cells with KU55933 (an ATM kinase‐specific inhibitor) and AZD6738 (an ATR kinase‐specific inhibitor). The results revealed that inhibiting p‐ATM significantly reduced the accumulation of the LC3B‐II/LC3B‐I ratio under CQ treatment and increased p62 expression (Figure 5C). In contrast, inhibiting p‐ATR did not have similar effects (Figure S5, Supporting Information). We then continued using KU55933 in T24 shXPC and 5637 shXPC cells. The results revealed a significant reduction in the accumulation of the number of RFP‐LC3B‐positive puncta and autophagosomes or autolysosomes (Figure 5D,E). In conclusion, these findings suggest that XPC knockdown promotes autophagy via p‐ATM kinase activation. To directly examine the impact of p‐ATM kinase activation on cisplatin sensitivity, we reassessed cisplatin sensitivity in XPC‐stably knockdown cell lines after inhibiting p‐ATM kinase activity via KU55933 treatment. CCK‐8 results demonstrated that the survival rate of KU55933‐treated cells under cisplatin stimulation was significantly reduced (Figure 5F). Colony formation assays indicated a marked decrease in colony formation rate (Figure 5G), suggesting that cisplatin sensitivity was significantly restored upon p‐ATM activity inhibition.

### 2.6 p‐ATM Activates Autophagy by Increasing KDM4A Expression

**Figure 6:**
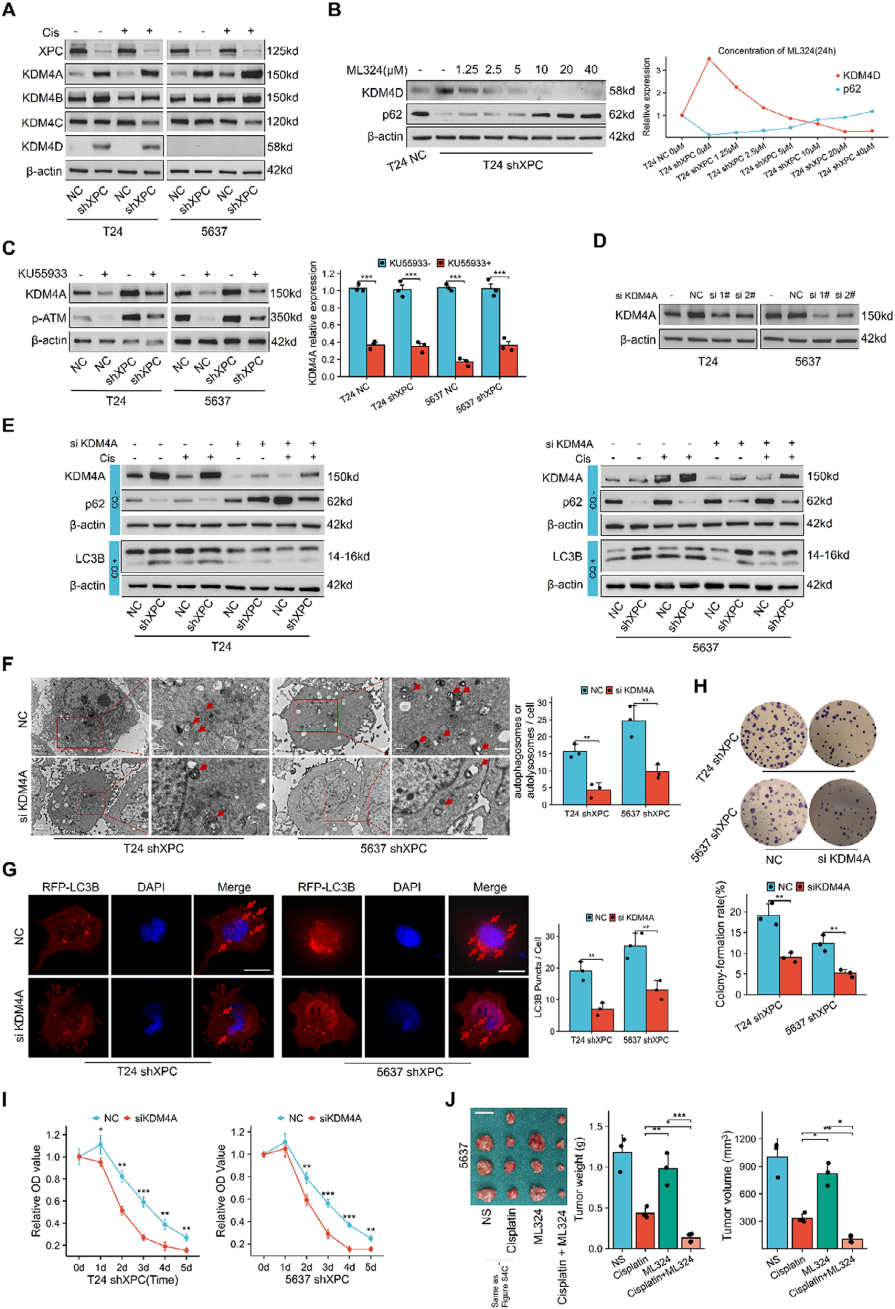
p‐ATM activates autophagy by increasing KDM4A expression. In vitro, we first examined the effects of XPC knockdown on the expression of KDM4 family proteins under basal conditions and upon cisplatin treatment (using the same conditions as previously described). KU55933 was consistently applied at 0.5 µm for 24 h. For the KDM4A rescue experiments using siKDM4A, cisplatin concentrations remained at 20 µm for T24 cells and 10 µm for 5637 cells, with total cellular proteins collected after 24 h (cisplatin exposure duration: 4 h). For Western blot analysis of LC3B, TEM examination, and RFP‐LC3B plasmid transfection experiments, CQ was employed as a control condition following the same treatment protocols as previously described. In vivo, the administration methods for cisplatin and ML324 are detailed in the Experimental Section. A) Western blot analysis of KDM4 family proteins in T24 and 5637 cells. B) Western blot analysis of KDM4D and p62 proteins in T24 shXPC cells after 24 h of treatment with varying ML324 concentrations. C) Western blot analysis of p‐ATM and KDM4A proteins in T24 and 5637 cells treated with KU55933 (0.5 µm) for 24 hours (*n* = 3). D) Western blot analysis of KDM4A protein levels in T24 and 5637 cells transfected with siKDM4A. E) Western blot analysis of KDM4A, LC3B, and p62 proteins in T24 and 5637 cells transfected with siKDM4A and treated with cisplatin for 24 h (T24: 20 µm; 5637: 10 µm). When detecting LC3B, 10 µm CQ treatment for 4 h was used as the control condition. F) TEM images of T24 shXPC and 5637 shXPC cells transfected with siKDM4A (*n* = 3), 10 µm CQ treatment for 4 h was used as the control condition. Scale bar: 1 µm. G) Fluorescence microscopy images of RFP‐LC3B puncta in T24 shXPC and 5637 shXPC cells transfected with siKDM4A (*n* = 3), 10 µm CQ treatment for 4 h was used as the control condition. Scale bar: 10 µm. H) Colony formation assay results for siKDM4A transfected T24 shXPC and 5637 shXPC cells stimulated with 20 µm cisplatin (T24) and 10 µm cisplatin (5637) (*n* = 3). I) CCK‐8 assay results for siKDM4A transfected T24 shXPC and 5637 shXPC cells after 1–5 d of stimulation with 20 µm cisplatin (T24) or 10 µm cisplatin (5637) (*n* = 3). J) Xenograft analysis of subcutaneous tumor growth in nude mice treated with cisplatin, ML324, or their combination (cisplatin: 5 mg/kg; ML324: 4 mg/kg, both intraperitoneally once per week) after 4 weeks. The data for the NS and cisplatin‐only groups were identical to those in Figure S4C in the Supporting Information. Scale bar: 1 cm. For each animal or independent cell culture experiment, measurements were repeated three times under the same experimental conditions. In each panel, dots represent individual measurements from animals or independent cell culture experiments. Bars represent group means, and error bars indicate ± standard deviation (SD). *p < 0.05, **p < 0.01, ***p < 0.001. Abbreviations: cis cisplatin, TEM Transmission electron microscopy, CQ chloroquine.

KDM4 histone demethylases are rapidly recruited to DNA damage sites during the DDR, where they phosphorylate downstream ATM substrates and promote double‐strand break repair.^[^ ^29^ ^]^ In yeast, the corresponding enzymes Rph1/KDM4 regulate autophagy during stress.^[^ ^30^ ^]^ Therefore, KDM4 may be a key mediator linking the DDR to autophagy. The Western blot results revealed that KDM4A was significantly upregulated after XPC knockdown. KDM4B changes were less pronounced, KDM4C expression was not significantly different, and KDM4D expression was undetectable in 5637 cells (**Figure** **6**A). ML324 is a well‐established KDM4 demethylase inhibitor.^[^ ^31^ ^]^ In T24 cells, as the ML324 concentration increased, the expression of p62 increased (Figure 6B). We then inhibited p‐ATM activity using KU55933. KDM4A expression decreased in parallel with p‐ATM reduction, regardless of XPC knockdown status (Figure 6C). We designed siRNAs targeting KDM4A. siKDM4A #1 had the best knockdown effect and was used for further experiments (Figure 6D). In XPC‐deficient cells, KDM4A knockdown reversed autophagy activation induced by XPC depletion (Figure 6E). Cells treated with siKDM4A presented significantly fewer autophagosomes or autolysosomes and RFP‐LC3B positive puncta (Figure 6F,G). These results indicate that p‐ATM induces autophagy via KDM4A upregulation. To directly investigate the effect of KDM4A overexpression on cisplatin sensitivity, we reassessed the response of the XPC stable knockdown cell line to cisplatin stimulation after transfection with siKDM4A. The CCK‐8 results showed that cell survival rate was significantly reduced under cisplatin stimulation after siKDM4A interference (Figure 6I). The colony formation assay indicated a significant decrease in colony formation rate (Figure 6H), suggesting that cisplatin sensitivity was markedly restored following the inhibition of KDM4A overactivation.

In a nude mouse tumor xenograft model, we assessed the effects of ML324 alone and combined cisplatin. The combination of cisplatin and ML324 strongly inhibited tumor growth, suggesting that KDM4A overactivation contributes to cisplatin resistance in bladder tumors (Figure 6J).

### 2.7 KDM4A Activates Autophagy by Suppressing PHRF1 Expression

**Figure 7:**
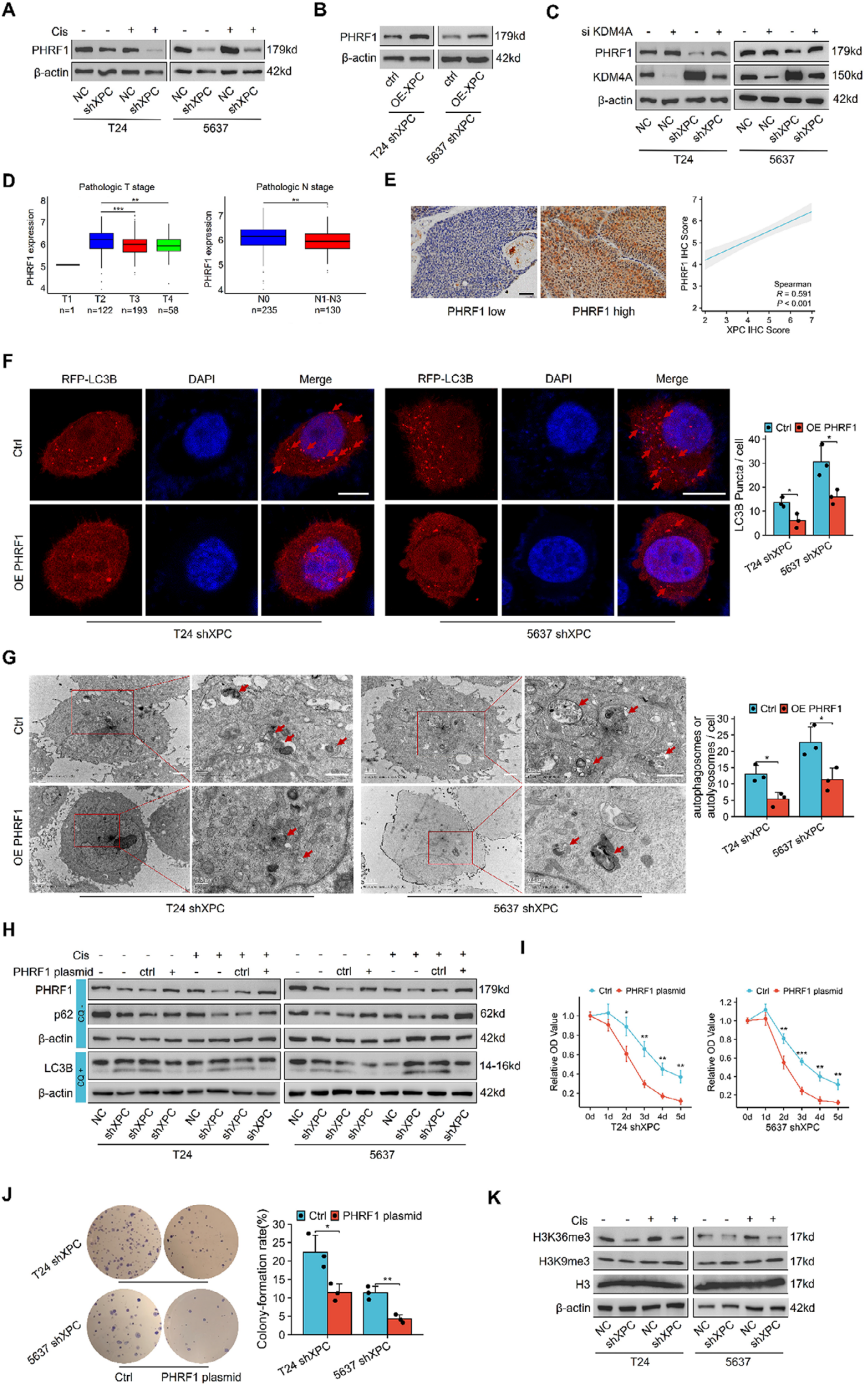
KDM4A activates autophagy by suppressing PHRF1 expression. In vitro, the cisplatin treatment conditions were consistent with those described previously. Protein expression of PHRF1 was examined under conditions of stable XPC knockdown and subsequent rescue. PHRF1 expression was re‐evaluated following KDM4A depletion via siRNA. Tissue microarray sections were subjected to immunohistochemical staining to analyze correlations between PHRF1 expression and clinical progression markers in bladder cancer patients, as well as its association with XPC expression levels. After constructing a eukaryotic PHRF1 overexpression plasmid and restoring PHRF1 expression, autophagy‐related proteins were reassessed. Additional validation included TEM and RFP‐LC3B plasmid transfection assays. For Western blot analysis of LC3B, TEM examination, and RFP‐LC3B plasmid transfection experiments, CQ was employed as a control condition following the same treatment protocols as previously described. A) Western blot analysis of PHRF1 protein levels in T24 and 5637 cells. B) Western blot analysis of PHRF1 protein levels in T24 shXPC and 5637 shXPC cells after XPC reintroduction using an XPC overexpression plasmid. C) Western blot analysis of PHRF1 protein levels in T24 and 5637 cells after siRNA‐mediated KDM4A knockdown. D) Statistical analysis of PHRF1 mRNA expression in bladder cancer patient data (including T stage, N stage) from the TCGA database. E) Representative immunohistochemical staining of PHRF1 in tissue microarray. Scale bar: 50 µm. Correlation analysis of PHRF1 and XPC expression levels in tissue microarrays from MIBC patients. F) Fluorescence microscopy images of RFP‐LC3B puncta in T24 shXPC and 5637 shXPC cells after PHRF1 plasmid introduction (*n* = 3). 10 µm CQ treatment for 4 h was used as the control condition. Scale bar: 10 µm. G) TEM images of T24 shXPC and 5637 shXPC cells after PHRF1 plasmid introduction (*n* = 3). 10 µm CQ treatment for 4 h was used as the control condition. Scale bar: 1 µm. H) Western blot analysis of PHRF1, LC3B, and p62 protein levels in T24 and 5637 cells after PHRF1 plasmid transfection and cisplatin treatment for 24 h (T24: 20 µm; 5637: 10 µm). When detecting LC3B, 10 µm CQ treatment for 4 h was used as the control condition. I) CCK‐8 assay results for PHRF1 plasmid introduced T24 shXPC and 5637 shXPC cells after 1–5 d of stimulation with 20 µm cisplatin (T24) or 10 µm cisplatin (5637) (*n* = 3). J) Colony formation assay results for PHRF1 plasmid introduced T24 shXPC and 5637 shXPC cells stimulated with 20 µm cisplatin (T24) and 10 µm cisplatin (5637) (*n* = 3). K) Western blot analysis of H3K36me3 and H3K9me3 protein levels in T24 and 5637 cells with or without cisplatin treatment. For each independent cell culture experiment, measurements were repeated three times under the same experimental conditions. In each panel, dots represent individual measurements from independent cell culture experiments. Bars represent group means, and error bars indicate ± standard deviation (SD). *p < 0.05, **p < 0.01, ***p < 0.001. Abbreviations: cis cisplatin, TEM Transmission electron microscopy, CQ chloroquine.

KDM4A contains a JmjN domain, a JmjC domain, two PHD domains, and two TUDOR domains.^[^ ^32^ ^]^ PHRF1 (PHD and ring finger domain 1) contains a PHD that binds to methylation modifications on histones and a zinc finger domain with E3 ubiquitin ligase activity.^[^ ^33^ ^]^ Recent studies have shown that ATM phosphorylates PHRF1 at S925 and S1389 in response to ionizing radiation‐induced DNA damage and that PHRF1 specifically interacts with H3K36 methylation sites.^[^ ^33^ ^]^ H3K36me3 is crucial in the CHK2‐mediated DNA damage repair pathway, where Rad53 (CHK2 homologue) regulates PHRF1 expression and Rph1 (KDM4 yeast homologue) dissociation during DNA damage.^[^ ^34^, ^35^ ^]^ PHRF1 protein levels in T24 and 5637 cells were significantly reduced following XPC knockdown (**Figure** **7**A). Additionally, restoring XPC expression resulted in increased PHRF1 levels (Figure 7B). PHRF1 expression significantly increased upon KDM4A inhibition, and the effect was more pronounced in XPC‐knockdown cells (Figure 7C).

PHRF1 mRNA data from bladder cancer patients in the TCGA database revealed a decrease in PHRF1 expression with tumor progression, including T stage, N stage (Figure 7D). Immunohistochemistry analysis of tumor tissue microarray revealed a strong positive relationship between PHRF1 and XPC expression (Figure 7E). We constructed a eukaryotic expression plasmid and confirmed its protein expression (Figure S6, Supporting Information). We reintroduced PHRF1 into XPC‐knockdown T24 and 5637 cells. Fluorescence microscopy revealed a significant reduction in the accumulation of RFP‐LC3B punctate, and transmission electron microscopy revealed a significant reduction in the number of autophagosomes or autolysosomes (Figure 7F,G). Additionally, the accumulation level of LC3B protein was significantly decreased, whereas the p62 level increased (Figure 7H). These results demonstrate that KDM4A induces autophagy by repressing PHRF1 expression. To directly examine the impact of PHRF1 expression on cisplatin sensitivity, we restored PHRF1 expression by transfecting PHRF1 plasmid into the XPC‐stably‐knockdown cell line and reassessed cellular response to cisplatin. CCK‐8 assay revealed that PHRF1‐transfected cells exhibited significantly reduced viability upon cisplatin treatment (Figure 7I). Colony formation assay demonstrated a marked decrease in colony formation rate (Figure 7J), indicating that restoration of PHRF1 expression significantly recovered cellular sensitivity to cisplatin. Further analysis in our cell model revealed that H3K9me3 levels remained unchanged, whereas H3K36me3 levels significantly decreased following XPC silencing (Figure 7K). Given that H3K36 methylation is associated with transcriptional activation, our results suggest that KDM4A may repress PHRF1 expression by removing H3K36 methylation at the PHRF1 promoter via classical epigenetic regulatory mechanisms.

### 2.8 PHRF1 Ubiquitinates and Degrades p53 to Regulate Autophagy

**Figure 8:**
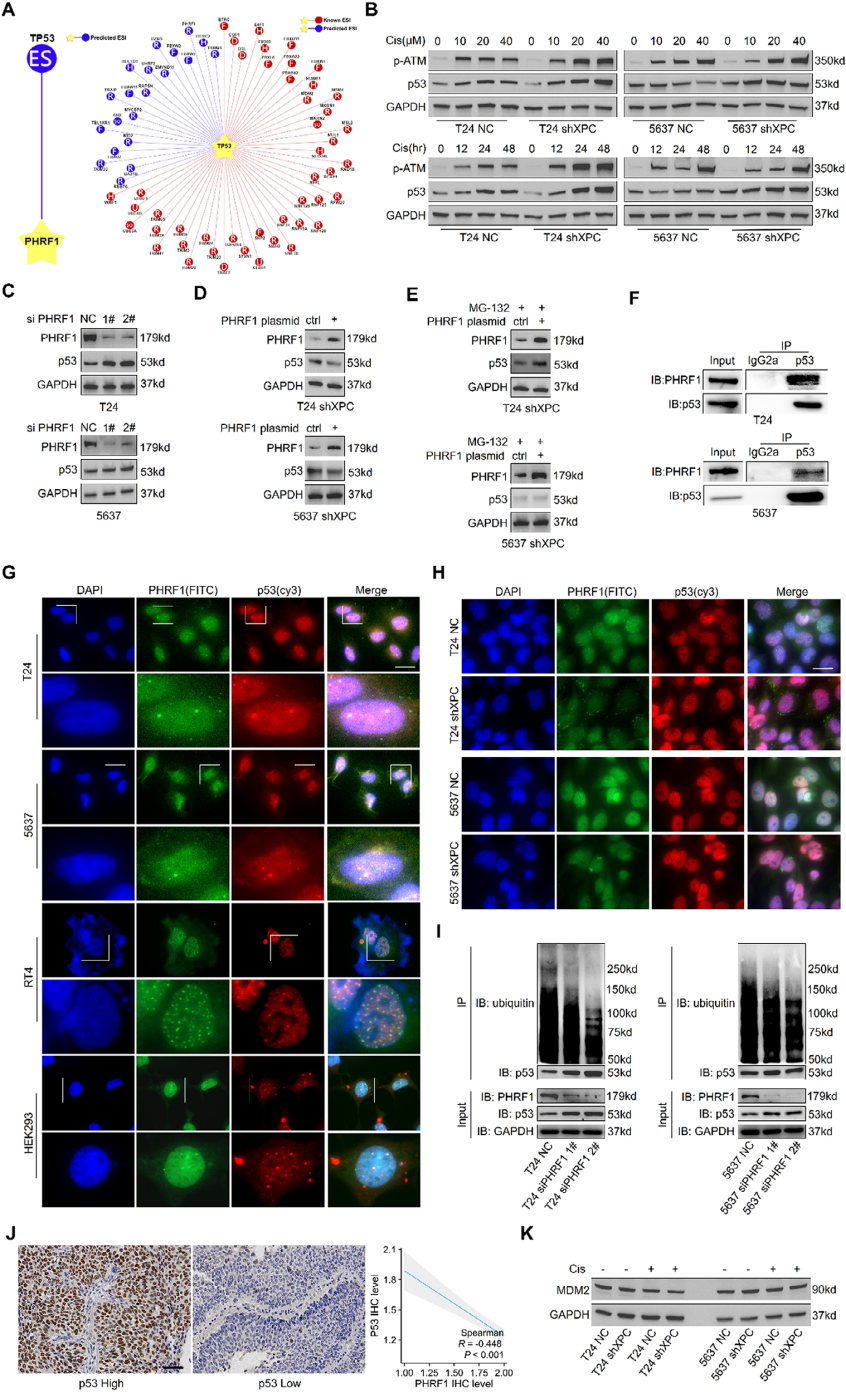
PHRF1 ubiquitinates and degrades p53 to regulate autophagy. In vitro, we first analyzed the differential expression of p53 protein in T24 and 5637shXPC cells after 24 h cisplatin treatment at varying concentrations compared to control groups. Subsequently, with fixed cisplatin concentration, we re‐examined p53 expression at different time points. Western blotting was performed to assess p53 protein changes in T24 and 5637 cells following PHRF1 knockdown via siRNA, followed by reevaluation of p53 expression after PHRF1 rescue using eukaryotic plasmids in T24 and 5637shXPC cells. After 8‐h treatment with 10 µm MG‐132, p53 expression changes were reassessed in PHRF1‐rescued T24 and 5637shXPC cells. Co‐IP was employed to examine potential interactions between PHRF1 and p53 proteins. Immunofluorescence colocalization experiments further investigated their spatial overlap in cells, with repeated colocalization assays post‐cisplatin treatment. Additional immunofluorescence assays determined PHRF1 spatial distribution in XPC‐knockdown cells. Co‐IP detection of ubiquitin‐conjugated p53 directly demonstrated reduced p53 ubiquitination levels upon PHRF1 knockdown. Western blotting evaluated MDM2 expression within this biological context. In vivo, immunohistochemical staining of tissue microarrays from bladder cancer patients was utilized to analyze the correlation between p53 and PHRF1 expression. Abbreviations: cis cisplatin. A) Prediction of PHRF1 ubiquitination substrates and p53 E3 ligases from the ubiquitination prediction database. B) Western blot analysis of the p53 protein in T24 and 5637 cells 24 h after cisplatin treatment (0–40 µm, removed after 4 h of treatment). Western blot analysis of the p53 protein in T24 and 5637 cells 0–48 h after cisplatin treatment. Cisplatin concentrations: 20 µm for T24 cells and 10 µm for 5637 cells. C) Western blot analysis of the PHRF1 and p53 proteins in wild‐type T24 and 5637 cells following siRNA‐mediated PHRF1 knockdown. D) Western blot analysis of the PHRF1 and p53 proteins in T24 shXPC and 5637 shXPC cells after the introduction of the PHRF1 plasmid. E) Western blot analysis of the PHRF1 and p53 proteins in T24 shXPC and 5637 shXPC cells after the introduction of the PHRF1 plasmid and treated with 10 µm MG‐132 for 8 h. F) Immunoprecipitation of p53 from T24 and 5637 cell lysates, followed by immunoblot analysis of input and IP samples. G) Immunofluorescence colocalization of PHRF1 and p53 under normal conditions in T24, 5637, RT4, and HEK293 cells. Scale bar: 20 µm. H) Immunofluorescence colocalization of PHRF1 and p53 in T24 and 5637 cells 24 h after cisplatin treatment. Cisplatin concentrations: 20 µm for T24 cells and 10 µm for 5637 cells. Scale bar: 20 µm. I) Immunoprecipitation of p53 followed by ubiquitin immunoblotting in T24 and 5637 cells 4 d after transfection with PHRF1‐targeting siRNA. J) Representative images of immunohistochemical staining of p53. Scale bar: 50 µm. Correlation between p53 and PHRF1 expression in human bladder cancer tissues. K) Western blot analysis of the MDM2 protein in T24 and 5637 cells 24 h after cisplatin treatment. Cisplatin concentrations: 20 µm for T24 cells and 10 µm for 5637 cells.

PHRF1, through its zinc finger domain, exhibits E3 ubiquitin ligase activity. We initially used the ubiquitination prediction database (http://ubibrowser.bio‐it.cn/ubibrowser_v3/) to identify potential PHRF1 substrates.^[^ ^36^, ^37^ ^]^ These results indicate that p53 is the primary substrate of PHRF1. Furthermore, by reverse‐predicting the likely E3 ligases for p53, PHRF1 was also identified (**Figure** **8**A). We initially examined p53 protein levels after XPC silencing. The results demonstrated that during the cisplatin‐induced DDR, p53 accumulated in a time‐ and dose‐dependent manner (Figure 8B). Moreover, PHRF1 knockdown in wild‐type T24 and 5637 cells resulted in marked accumulation of the p53 protein (Figure 8C). As both T24 and 5637 cells have mutated TP53, we expanded the experiments to include RT4 (a TP53 wild‐type bladder cancer line) and HEK293 cells. Similar results were observed for p53 protein accumulation after PHRF1 knockdown (Figure S7, Supporting Information). The reintroduction of PHRF1 via a eukaryotic plasmid partially rescued p53 protein levels (Figure 8D). However, treatment with MG‐132, a proteasome inhibitor, abolished the ability of the PHRF1 plasmid to restore p53 levels (Figure 8E).

To assess whether PHRF1 ubiquitinates p53 directly, we conducted coimmunoprecipitation (co‐IP) analysis and found that endogenous PHRF1 interacts with p53 in T24 and 5637 cells (Figure 8F). Immunofluorescence colocalization analysis confirmed that under normal conditions, both p53 and PHRF1 localize mainly to the nucleus, where they exhibit significant spatial overlap, indicating potential interactions (Figure 8G). However, upon cisplatin treatment, the overlap between PHRF1 and p53 in the nucleus was significantly reduced, suggesting that p53 accumulation during DDR may result from disrupted PHRF1‐p53 interactions (Figure S8, Supporting Information). Further analysis of XPC‐knockdown T24 and 5637 cells treated with cisplatin revealed reduced PHRF1 expression and PHRF1 translocation from the nucleus to the cytoplasm (Figure 8H). This not only reduces the overall expression of PHRF1 but also sequesters it away from the majority of nuclear p53, preventing its interaction, which may be the key reason for the massive accumulation of the p53 protein. These results also indicate that p53 accumulation due to XPC knockdown primarily occurs in the nucleus. Numerous studies have confirmed that the nuclear accumulation of p53 during stress responses is a potent inducer of autophagy.^[^ ^38^, ^39^, ^40^ ^]^ To evaluate p53 polyubiquitination, we performed immunoprecipitation and found that PHRF1 knockdown using two distinct siRNAs significantly reduced p53 polyubiquitination levels (Figure 8I). Immunohistochemical analysis of tumor tissues revealed a significant inverse correlation between PHRF1 and p53 expression levels (Figure 8J). We also assessed the status of MDM2, a well‐characterized E3 ligase for p53. MDM2 expression was unaffected by XPC status or cisplatin treatment, indicating that MDM2 does not play a major regulatory role in our model (Figure 8K). We further evaluated the prognostic impact of MDM2 in bladder cancer patients, but no statistically significant association was observed. In contrast, low PHRF1 expression significantly reduced OS in MIBC patients (Figure S9, Supporting Information). We examined the potential causes of this phenomenon in the discussion.

The Human Tumor Genome Project revealed that while germline TP53 mutations are extremely rare in bladder cancer patients (0.008%), somatic TP53 alterations (including 49% mutations and 21% copy number variations) occur in up to 70% of bladder tumor tissues.^[^ ^41^, ^42^ ^]^ We stratified bladder cancer patients based on XPC expression levels and p53 mutation status. Survival analysis demonstrated that patients with low XPC expression combined with p53 mutations exhibited the poorest clinical prognosis, with significantly shorter survival times compared to other subgroups (Figure S10, Supporting Information). Consistently, analysis of TCGA data showed that XPC expression was markedly reduced in TP53 mutant bladder cancers compared to TP53 wild type tumors (Figure 1J). These findings suggest a regulatory role of XPC on p53 protein and highlight the synergistic effect of XPC deficiency and TP53 mutations in bladder cancer progression.

## 3 Discussion

Our previous study indicates that XPC deficiency frequently occurs in human bladder cancer and significantly impacts tumor prognosis.^[^ ^7^, ^16^ ^]^ In this study, we demonstrated that reduced XPC expression promotes autophagy and cisplatin resistance in bladder cancer cells, a role distinct from the classical DNA damage recognition function of XPC. Thus, we revealed a novel XPC‐mediated signaling pathway that regulates autophagic flux and chemotherapeutic sensitivity. Additionally, through mechanistic studies, we identified a novel E3 ubiquitin ligase that targets p53.

Friedberg reported that XPC knockout mice are more prone to developing solid malignancies (skin, liver, lung, bladder tumors, etc.) when exposed to various carcinogenic factors.^[^ ^43^, ^44^ ^]^ Recent studies reported that autophagy‐deficient mouse embryonic fibroblasts from Atg5 knockout mice presented significantly impaired NER capacity and reduced XPC expression, providing direct evidence that autophagy is involved in NER regulation.^[^ ^45^ ^]^ The interplay between the DDR and autophagy largely determines cell fate and affects cellular behavior.^[^ ^46^ ^]^ We observed that under cisplatin stimulation, XPC‐deficient bladder cancer cells exhibited significantly increased autophagy, suggesting that DDR‐related genes are involved in the regulation of autophagy. However, XPC's DDR‐related function does not seem to dominate this regulation, as the knockdown of other key DDR genes (e.g., XPF and XPA) does not affect autophagy. If XPC's DDR‐related functions are primarily responsible, tumor cells are expected to become more sensitive to cisplatin and other chemotherapeutics due to an impaired DDR process. However, the opposite is true, which prompted us to investigate this further.

Numerous studies have revealed that autophagy in tumor cells promotes drug resistance, invasion, and metastasis.^[^ ^47^, ^48^, ^49^ ^]^ Recent studies have shown that KDM4 overexpression disrupts the DNA mismatch repair pathway.^[^ ^50^ ^]^ Clinically, elevated KDM4 and reduced H3K9/H3K36 methylation are linked to poor survival and promote gastrointestinal stromal tumor progression.^[^ ^51^ ^]^ KDM4 hyperactivation is also closely associated with stemness maintenance in various types of stem cells, including hematopoietic and triple‐negative breast cancer stem cells.^[^ ^52^, ^53^ ^]^ These findings highlight the role of KDM4 in DNA repair, autophagy, and cancer prognosis. We found that cisplatin combined with ML324 was more effective in XPC‐deficient cells than in those with normal XPC expression. This may be due to synthetic lethality between XPC and KDM4 in bladder cancer cells. This phenomenon may present a new therapeutic strategy for treating bladder cancer patients with XPC defects. Our previous research revealed that XPC knockout mice aged significantly faster than did wild‐type mice. In this study, we found that XPC knockdown in bladder cancer cells significantly reduced their proliferation rate. These findings suggest that XPC deficiency may contribute to senescence‐associated secretory phenotype (SASP)‐induced changes in the tumor microenvironment and the worsening of malignancy. Matthew reported that autophagy protects ageing hematopoietic stem cells by maintaining self‐renewal and differentiation while safeguarding against external stress, and this process occurs more readily as cells age.^[^ ^54^ ^]^ In most normal somatic cells, autophagy decreases with age; however, in cancer cells, particularly cancer stem cells, the opposite may occur. This may explain the observed increase in the number of bladder cancer stem cells after XPC knockdown.

PHRF1 first attracted attention because of its unique primary structure. PHRF1 has E3 ubiquitin ligase activity, and through database prediction and experimental validation, we identified p53 as a major substrate of PHRF1. We found that p53 accumulation after XPC knockdown is due primarily to reduced PHRF1 expression rather than alterations in MDM2 regulation. Additionally, IF assays revealed that XPC knockdown mislocalizes PHRF1 from the nucleus to the cytoplasm. This not only reduces the overall expression of PHRF1 but also sequesters it away from the majority of nuclear p53, preventing its interaction, which may be the key reason for the massive accumulation of the p53 protein. However, the mechanism behind PHRF1 nuclear export remains unclear. In our models, the regulatory effect of MDM2 on p53 ubiquitination was significantly weaker than that of PHRF1. Notably, Krzeszinski reported that p53 protein degradation was impaired in XPC‐knockdown cells, a phenomenon independent of the NER function of XPC.^[^ ^55^ ^]^ Additionally, overexpressing MDM2 did not reduce p53 levels in XPC‐deficient cells.^[^ ^55^ ^]^ These insights suggest that PHRF1 may play a more prominent role than MDM2 in the posttranslational regulation of p53 in specific biological contexts.

Classical studies have established that DNA damage induces phosphorylation of MDM2 (Ser395) via the ATM/CHK2 pathway, leading to its inactivation and loss of ubiquitin‐mediated degradation of p53.^[^ ^56^, ^57^ ^]^ In this study, we demonstrate that XPC deficiency strongly activates the ATM/CHK2 pathway, and this effect is further amplified by cisplatin‐induced DNA damage, resulting in hyperactivation of functional ATM in XPC‐low MIBC cell lines. This may explain the inability of MDM2 to ubiquitinate and regulate p53 protein under these biological conditions. From a clinical perspective, Lyubomir T. et al. demonstrated through in vivo studies that Nutlin‐3 (a small‐molecule MDM2 antagonist) exhibits significant antitumor activity in wild‐type p53 tumor models.^[^ ^58^ ^]^ However, subsequent studies revealed that in tumors with p53 mutations or deletions, MDM2 inhibitors (such as Nutlin‐3) fail to activate the apoptotic pathway, leading to markedly reduced efficacy, particularly in cisplatin‐resistant cells, where MDM2 inhibitors were ineffective.^[^ ^59^ ^]^ In this context, our study identifies PHRF1 as a novel regulator capable of ubiquitinating and degrading mutant p53 protein, even under cisplatin‐induced DNA damage. Furthermore, in the survival analysis of bladder cancer patients from this cohort, PHRF1 was identified as a strong independent prognostic factor. This discovery provides new insights into the complexity of p53 regulation in the DDR and suggests PHRF1 as a potential therapeutic target. Given that TP53 mutations occur in up to 70% of bladder cancer cases, this finding holds significant translational potential and warrants further exploration.

Recent advances in tumor‐targeted therapies have revealed that while no drugs directly target p53, more than twenty compounds targeting the p53‐specific ubiquitin ligase MDM2 are in clinical trials, making this a hot topic in cancer drug development. Our study presents an alternative target to MDM2 with broader clinical potential. PHRF1 strongly regulates p53 posttranslationally and modulates the response of bladder cancer cells to chemotherapy. Additionally, in collaboration with the University of Gothenburg, we observed similar phenomena in three pancreatic cancer cell lines.

In conclusion, our study reveals XPC's nonclassical role in DNA damage repair and highlights its upstream signaling functions. We emphasize the critical roles of XPC and its downstream components, PHRF1 and KDM4A, in regulating autophagy and chemotherapeutic sensitivity in bladder cancer. Additionally, we identified a novel p53 ubiquitin ligase that may serve as a therapeutic target in bladder cancer.

## 4 Experimental Section

### Animals

XPC knockout (KO) mice (B6;129‐XPC^1Ecf/^J) were purchased from Jackson Laboratory, and nude mice were obtained from Charles River Laboratories; all the mice were female and 4–5 weeks old. All animals were housed in a specific pathogen‐free (SPF) environment at the Experimental Animal Center of Army Medical University; enrichment materials included nesting materials and dental blocks. All experimental protocols were approved by the Animal Ethics Committee of Army Medical University (Approval Number: AMUWEC20232984), ensuring strict adherence to relevant animal welfare measures. All procedures and research complied with international and Chinese guidelines and regulations.

### Plasmids, Lentiviruses, Oligonucleotides, and Cell Lines

The XPC plasmid was constructed as described in our previous publications,^[^ ^8^ ^]^ the PHRF1 plasmid was constructed and validated by a commercial company, and the RFP‐LC3B plasmid was kindly provided by J. Lian, an expert in autophagy research at our university. The XPC knockdown lentivirus was obtained from a commercial company. KDM4A and PHRF1 siRNAs were synthesized and dual‐validated by a commercial company. The HEK293(RRID: CVCL_0045), T24(RRID: CVCL_0554), 5637(RRID: CVCL_0126), SW1710(RRID: CVCL_1721), and RT4 (RRID: CVCL_0036) cell lines were sourced from the Cell Bank of the Chinese Academy of Sciences in 2019. The maximum number of passages for these cell lines was 20, and mycoplasma testing was conducted regularly to ensure the free of mycoplasma contamination. All the cell lines were cultured following the supplier's instructions. Key material information was provided in Table S1 in the Supporting Information.

### Cancer Tissue Samples

Tumor tissues and adjacent nontumor tissues were collected from 90 patients with MIBC who had not received radiotherapy or chemotherapy at Southwest Hospital, affiliated with Army Medical University. All patients included in this study had pathologically confirmed MIBC postsurgery, and none had severe comorbidities, such as other malignant tumors or significant cardiovascular or cerebrovascular diseases. The study was conducted according to the guidelines of the Declaration of Helsinki and approved by the Ethics Committee of Southwest Hospital (Approval Number: KY2020157). This study has obtained informed consent from all patients for the use of their clinical samples and data in this research.

### Tumor Xenograft Implantation Experiment

The tumor xenograft implantation experiment was conducted as previously described.^[^ ^60^ ^]^ Briefly, 1 × 10^6^ T24 or 5637 cells were subcutaneously injected into the axillary region of female nude mice aged 4–5 weeks. Tumor growth was monitored daily. After approximately two weeks, once stable xenograft growth was established, the mice were divided into groups for different drug treatments. Cisplatin was administered via intraperitoneal injection at a dose of 5 mg/kg body weight once a week;^[^ ^61^ ^]^ CQ was administered at 80 mg/kg body weight once daily;^[^ ^62^ ^]^ and ML324 was administered at 4 mg/kg body weight once a week. Changes in tumor size and the survival status of the mice were continuously observed and recorded throughout the treatment period. After six weeks, the experiment was terminated. The tumors were collected, weighed, and analyzed further. Key material information was provided in Table S1 in the Supporting Information.

### CCK‐8 and Colony Formation Assays

A Cell Counting Kit‐8 (CCK‐8) assay was used to evaluate drug sensitivity. Briefly, 1000–5000 cells were seeded into 96‐well plates; replicates were prepared for each condition. Drug treatments were administered via either a time gradient or a concentration gradient. The absorbance at 450 nm was measured via a spectrophotometer following the manufacturer's instructions for the CCK‐8 assay. The colony formation assay was conducted as previously described.^[^ ^60^ ^]^ Briefly, 500–1000 cells were seeded into each well of a 6‐well plate and cultured for 7–14 d. After colony formation, the plates were washed with phosphate‐buffered saline (PBS), fixed with 4% paraformaldehyde, and stained with crystal violet, after which the colonies were counted; the data were statistically analyzed. Key material information was provided in Table S1 in the Supporting Information.

### Immunohistochemistry (IHC) and Immunofluorescence (IF)

IHC and IF were conducted according to standardized procedures as previously described.^[^ ^63^ ^]^ IHC images were acquired via an Olympus optical microscope and analyzed with Olympus OlyVIA software. Quantification was performed via the classic immunohistochemical scoring system. For the IF experiments, which included observation of the RFP‐LC3B plasmid results, images were captured via a Zeiss fluorescence microscope (Axio Imager M2). Key material information was provided in Table S1 in the Supporting Information.

### Western Blotting

Equal amounts of cell lysates (20–50 µg of total protein) were separated via SDS‐PAGE and subsequently analyzed by Western blotting, following previously described procedures.^[^ ^8^ ^]^ The details of the antibodies utilized were provided in the Table S1 in the Supporting Information.

### Transmission Electron Microscopy (TEM)

The preparation and observation of the TEM samples were conducted with assistance from the staff of the Electron Microscope Room at the Central Laboratory of Army Medical University. TEM images were acquired using a TECNAI 10 electron microscope (Philips, Netherlands).

### Coimmunoprecipitation (co‐IP)

Cells were lysed in specialized IP lysis buffer supplemented with protease inhibitors to extract total protein following previously described procedures.^[^ ^64^ ^]^ Coimmunoprecipitation was conducted according to the manufacturer's instructions for the co‐IP kit. Key material information was provided in Table S1 in the Supporting Information.

### Sphere Culture

The bladder cancer cell suspension sphere formation assay was conducted following a previously published protocol.^[^ ^65^ ^]^ Each 100 mL of sphere‐forming medium comprised 97 mL of serum‐free medium, 2 mL of B27, 2 µg of EGF, 2 µg of bFGF, 500 µg of insulin, and 1 mL of penicillin/streptomycin solution. Well‐adherent cells in good condition were enzymatically digested with trypsin, centrifuged at 800 rpm to remove the supernatant, and then resuspended in PBS. This washing step was repeated twice. After cell counting, 10 000 cells per well were seeded in a 6‐well ultralow attachment culture plate. The cells were cultured for 5–14 d, after which they were monitored daily for sphere formation. Once spheres formed, they were transferred to a 15 mL centrifuge tube and collected after gravity settling for subsequent experiments. The sphere formation efficiency (SFE) was calculated as follows: SFE = (number of spheres with a diameter > 75 µm per well) / (total number of originally seeded cells per well).

### 3D Cell Explore

Cells were plated in 35 mm confocal dishes, and live cell morphological changes were recorded following the manufacturer's instructions (3D Cell Explorer, Nanolive, Switzerland).

### Statistical Analyses

All the data were analyzed via R software (version 4.2.1) or GraphPad Prism software (version 8.4.0). The data are expressed as the means ± standard deviations (SDs). Intergroup differences were assessed via Student's *t* test or the Wilcoxon rank‐sum test via the ggplot2 package. Survival probabilities were estimated via the Kaplan–Meier method (log‐rank test or cox regression model) with the survminer and survival packages. Chi‐square tests were used to analyze categorical data. A *P* value of less than 0.05 was considered to indicate statistical significance.

## Conflict of Interest

The authors declare no conflict of interest.

## Supporting information



Supporting Information

## Data Availability

The data that support the findings of this study are available from the corresponding author upon reasonable request.
