# Supplementary material for: XPC Deficiency Activate Cisplatin‐Mediated Autophagy in Bladder Cancer by Limiting Novel PHRF1‐Mediated Ubiquitination of the p53 Protein
Source: Adv Sci (Weinh). 2025 Nov 11;13(5):e17563. doi: 10.1002/advs.202517563 (PMC12850319; doi:10.1002/advs.202517563)
Supplement: Supplementary file 1 — Supporting Information [file ADVS-13-e17563-s001.docx]

Supporting Information

**XPC Deficiency Activate Cisplatin-mediated Autophagy in Bladder Cancer by Limiting Novel PHRF1-mediated Ubiquitination of the p53 Protein**

*Baixiong Zhao*, *Yaqin Huang*, *Jiazhong Shi*, *Xiaozhou Zhou*, *Johan Bourghardt Fagman*, *Liwei Wang*, *Sha Liu*, *Wuxing Wang*, *Yuting Liu*, *Zhiwen Chen*,^*^ *and Jin Yang*^*^

**Figure S1 Page 2**

**Figure S2 Page 2**

**Figure S3 Page 3**

**Figure S4 Page 4**

**Figure S5 Page 5**

**Figure S6 Page 5**

**Figure S7 Page 5**

**Figure S8 Page 6**

**Figure S9 Page 7**

**Figure S10 Page 7**

**Table S1 Page 8**


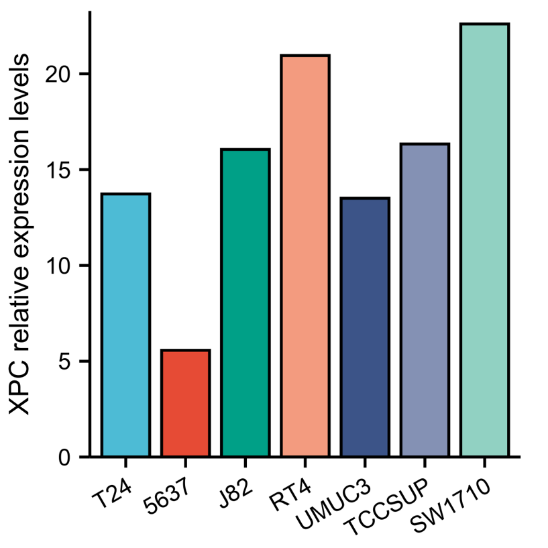


Figure S1. Analysis of the relative expression levels of XPC in common human bladder cancer cell lines based on the CCLE database.


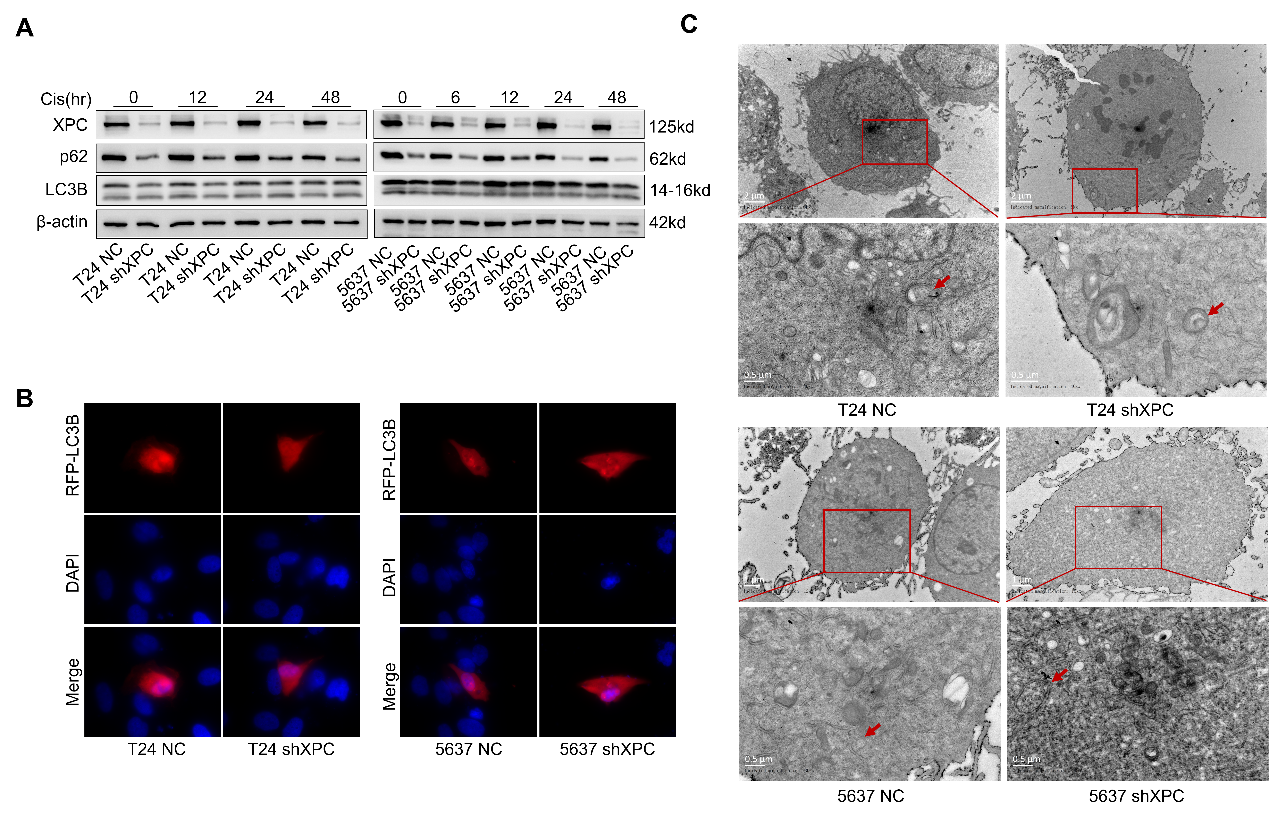


Figure S2. Under basal conditions (without CQ treatment), detection of autophagy-related proteins, transmission electron microscopy images of autophagosomes or autolysosomes, and fluorescence microscopy images of RFP-LC3B plasmid-positive puncta in T24 and 5637 cells following XPC knockdown. Cisplatin was maintained at concentrations of 20 μm for T24 cells and 10 μm for 5637 cells (removed after 4 hours of treatment).


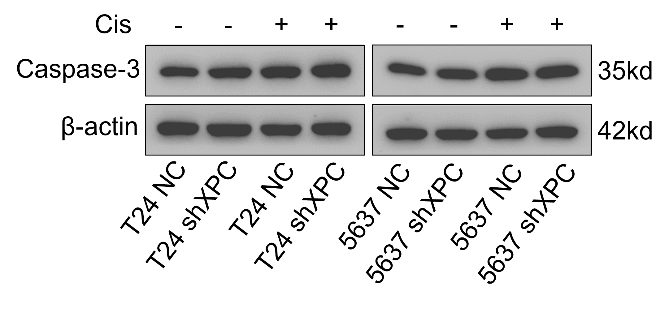


Figure S3. Western blot analysis of caspase-3 proteins in T24 and 5637 cells.


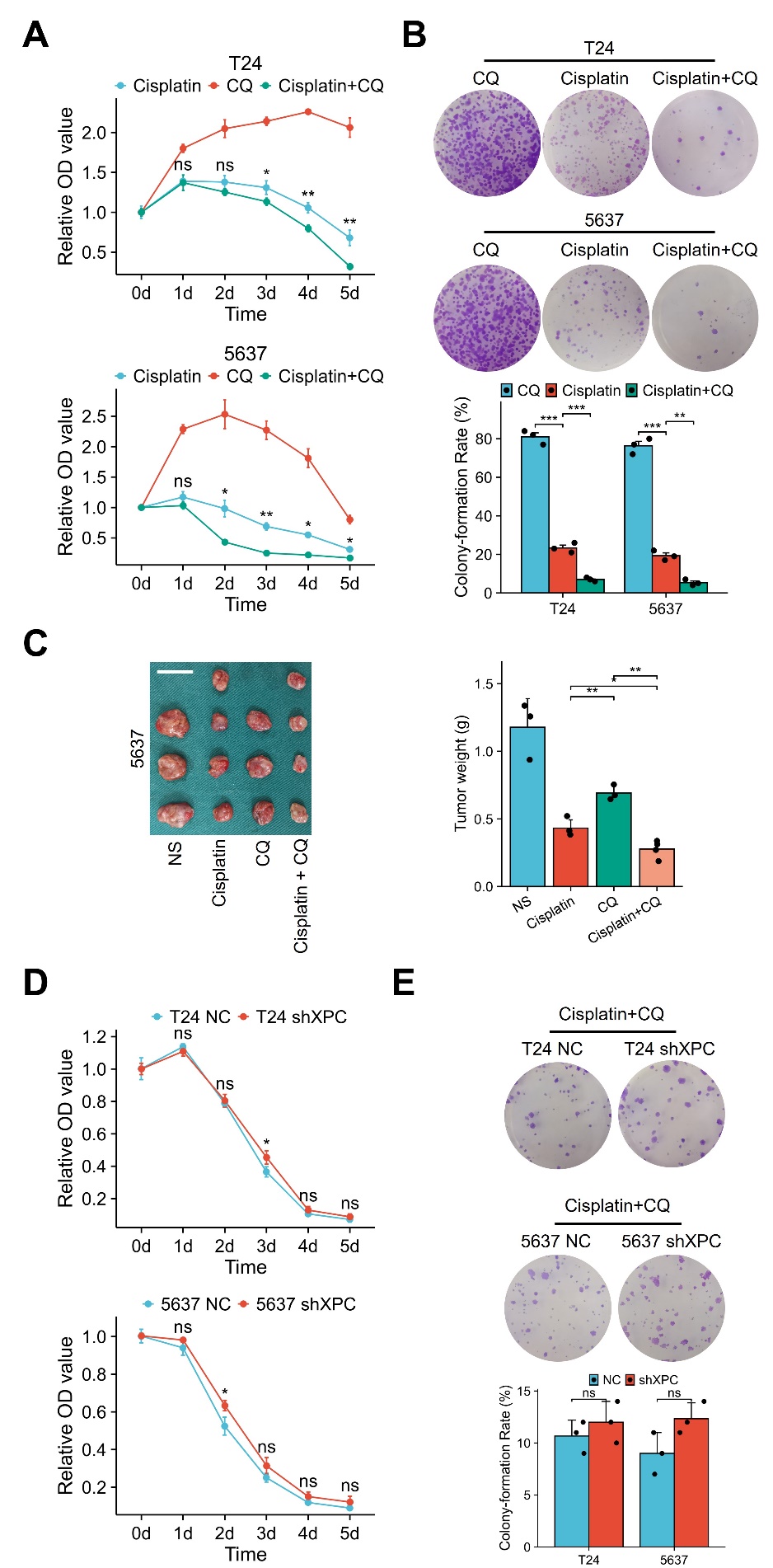


Figure S4. XPC knockdown in bladder cancer cells induces cisplatin resistance via autophagy activation.

(A) CCK-8 assay results for T24 and 5637 cells treated with cisplatin, CQ, or their combination for 1-5 days. T24 cells were treated with 20 μm cisplatin, 5637 cells were treated with 10 μm cisplatin, and both cell lines were treated with 20 μm CQ.

(B) Colony formation assay results for T24 and 5637 cells treated with cisplatin, CQ, or their combination. The drug concentrations were identical to those mentioned above (*n*=3).

(C) Tumor weights of subcutaneous xenografts in nude mice after 4 weeks of treatment with cisplatin (5 mg/kg, once per week, intraperitoneally), CQ (80 mg/kg, once daily, intraperitoneally), or their combination. Scale bar: 1 cm.

(D) CCK-8 assay results for T24 and 5637 NC and shXPC cells treated with 20 μm CQ for 1-5 days. T24 cells were treated with 20 μm cisplatin, and 5637 cells were treated with 10 μm cisplatin.

(E) Colony formation assay results for T24 and 5637 NC and shXPC cells treated with cisplatin and 20 μm CQ. T24 cells were treated with 20 μm cisplatin, and 5637 cells were treated with 10 μm cisplatin (*n*=3).


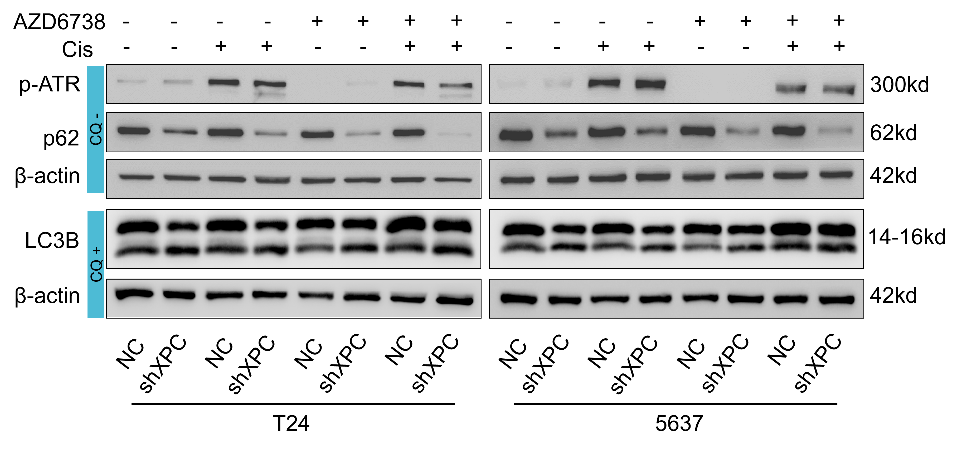


Figure S5. Western blot analysis of p-ATR, LC3B, and p62 protein levels in T24 and 5637 cells after 24-hour treatment with AZD6738 (0.1 μm) and cisplatin (20 μm for T24 cells, 10 μm for 5637 cells).


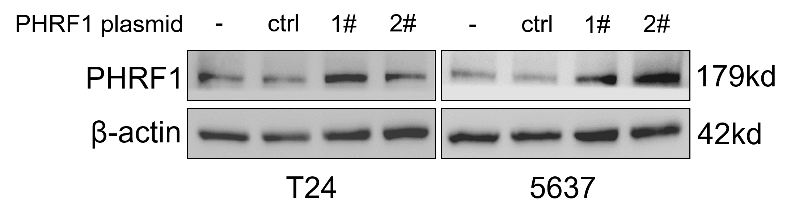


Figure S6. Western blot analysis of PHRF1 protein levels in T24 and 5637 cells after PHRF1 overexpression plasmid introduction.


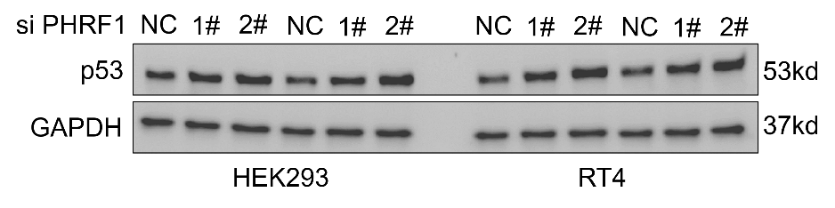


Figure S7. Western blot analysis of p53 proteins in wild-type HEK293 and RT4 cells following siRNA-mediated PHRF1 knockdown.


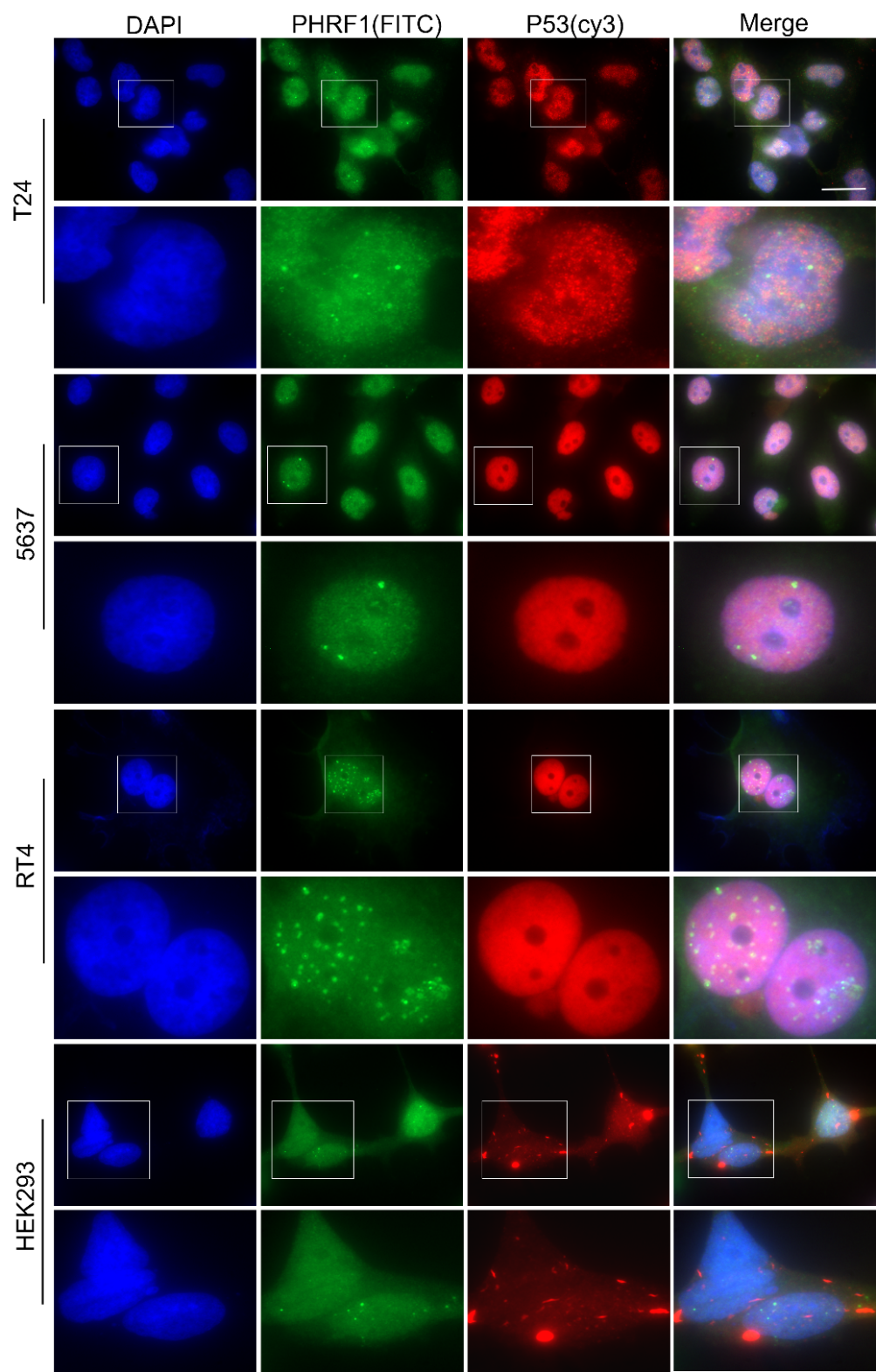


Figure S8. Immunofluorescence colocalization of PHRF1 and p53 in T24, 5637, RT4, and HEK293 cells 24 hours after cisplatin treatment. Cisplatin concentrations: 20 μm for T24 and RT4 cells, 10 μm for 5637 and HEK293 cells.


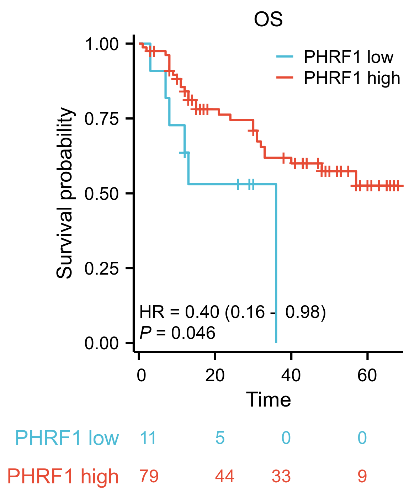


Figure S9. Survival analysis of MIBC patients stratified by PHRF1 expression levels in MIBC cases.


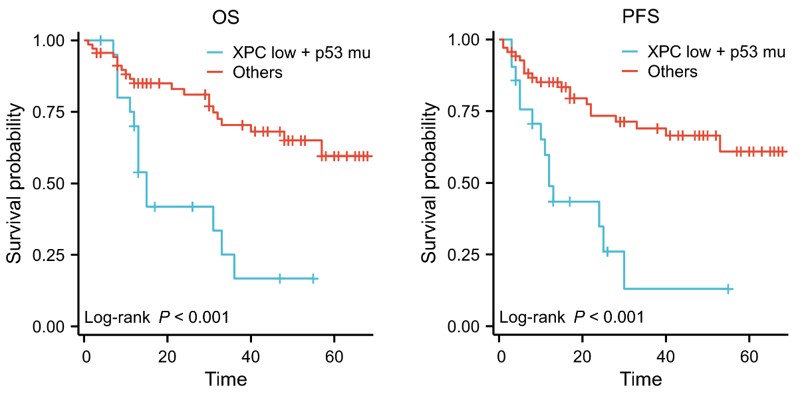


Figure S10. Analysis of survival differences between XPC low + p53 mutant patients and the remaining cohort in MIBC cases.

Table S1. Key material information

| Reagent or Resource | Source | Identifier |
| --- | --- | --- |
| Antibodies |  |  |
| Mouse monoclonal anti-XPC | GeneTex | Cat# GTX70294; RRID: AB_373299 |
| Mouse monoclonal anti-CD44(8E2) | Cell Signaling Technology | Cat# 5640; RRID: AB_10547133 |
| Rabbit monoclonal anti-β-Actin (13E5) | Cell Signaling Technology | Cat #4970; RRID:AB_2223172 |
| Mouse monoclonal anti- P62,SQSTM1 | Proteintech | Cat #66184-1-Ig; RRID:AB_2881579 |
| Rabbit polyclonal anti-LC3B | Sigma-Aldrich | Cat # L7543; RRID:AB_796155 |
| Rabbit monoclonal anti- ATM (D2E2) | Cell Signaling Technology | Cat# 2873; RRID:AB_2062659 |
| Rabbit monoclonal anti-p-ATM (Ser1981) (D6H9) | Cell Signaling Technology | Cat# 5883; RRID:AB_10835213 |
| Rabbit monoclonal anti- ATR (E1S3S) | Cell Signaling Technology | Cat# 13934; RRID:AB_2798347 |
| Rabbit monoclonal anti-p-ATR (Thr1989) (D5K8W) | Cell Signaling Technology | Cat# 2853; RRID:AB_2290281 |
| Rabbit monoclonal anti-p- Chk2 (Thr68) (C13C1) | Cell Signaling Technology | Cat# 2197; RRID:AB_2080501 |
| Rabbit monoclonal anti- KDM4A / JHDM3A / JMJD2A | Abcam | Cat# ab191433 |
| Rabbit monoclonal anti-KDM4B / JMJD2B (D7E6) | Cell Signaling Technology | Cat# 8639; RRID:AB_11140642 |
| Rabbit polyclonal anti- KDM4C / GASC1 / JMJD2C | Abcam | Cat# ab85454; RRID:AB_2129195 |
| Rabbit polyclonal anti- KDM4D / JMJD2D | Abcam | Cat# ab93694; RRID:AB_10561782 |
| Rabbit polyclonal anti-PHRF1 | Abcam | Cat# ab85974; RRID:AB_1925150 |
| Rabbit monoclonal anti- Histone H3 (D1H2) | Cell Signaling Technology | Cat# 4499; RRID:AB_10544537 |
| Rabbit monoclonal anti-Tri-Methyl-Histone H3 (Lys9) (D4W1U) | Cell Signaling Technology | Cat# 13969; RRID:AB_2798355 |
| Rabbit monoclonal anti-Tri-Methyl-Histone H3 (Lys36) (D5A7) | Cell Signaling Technology | Cat# 4909; RRID:AB_1950412 |
| Rabbit monoclonal anti-GAPDH | ABclonal | Cat# A19056; RRID:AB_2862549 |
| Mouse monoclonal anti-p53 (DO-1) | Santa Cruz Biotechnology | Cat# sc-126; RRID:AB_628082 |
| Mouse monoclonal anti- Ubiquitin(P4D1) | Santa Cruz Biotechnology | Cat# sc-8017; RRID:AB_628423 |
| Rabbit monoclonal anti- MDM2 (D1V2Z) | Cell Signaling Technology | Cat# 86934; RRID:AB_2784534 |
| Rabbit monoclonal anti-Sox2 (D9B8N) | Cell Signaling Technology | Cat# 23064; RRID:AB_2714146 |
| Rabbit monoclonal anti- Nanog (D73G4) | Cell Signaling Technology | Cat# 4903; RRID:AB_10559205 |
| Mouse monoclonal anti-Oct-4 (D7O5Z) | Cell Signaling Technology | Cat# 75463; RRID:AB_2799870 |
| Rabbit monoclonal anti- Caspase-3 (D3R6Y) | Cell Signaling Technology | Cat# 14220; RRID:AB_2798429 |
| Chemicals | | |
| Cisplatin | MedChem Express | Cat# HY-17394 |
| KU-55933 | MedChem Express | Cat# HY-12016 |
| ML324 | MedChem Express | Cat# HY-12725 |
| Ceralasertib (Synonyms: AZD6738) | MedChem Express | Cat# HY-19323 |
| Chloroquine (Synonyms: CQ) | MedChem Express | Cat# HY-17589A |
| MG-132 | MedChem Express | Cat# HY-13259 |
| Critical commercial assays | | |
| DAB substrate kit | Absin | Cat# abs9210-1kit |
| Cell Counting Kit-8 | BIOGROUND | Cat# BG0025 |
| Crystal Violet Staining Solution | Beyotime | Cat# C0121 |
| Super ECL Plus Western Blotting Substrate | BIOGROUND | Cat# BG0001 |
| Pierce™ Direct Magnetic IP/Co-IP Kit | Thermo Fisher Scientific | Cat# 88828 |
| RIPA Lysis Buffer | Beyotime | Cat# P0013B |
| TGX Stain-Free™ FastCast™ Acrylamide Kit | Bio-rad | Cat# 1610183; Cat# 1610185; Cat# 1610181 |
| Lipofectamine™ 2000 | Thermo Fisher Scientific | Cat# 11668019 |
| Matrigel® Basement Membrane | Corning | Cat# 354248 |
| Experimental models: Organisms | | |
| Mouse: XPC KO (B6; 129-Xpctm1Ecf/J) | Jackson Laboratory | N/A |
| Mouse: Nude Mice Nu/Nu | Charles River | Cat# 403 |
| RNA interference target site(5'-3') | | |
| shNC: GAAGAGAAGACAACGAACAGA | Tsingke Biotech | N/A |
| shXPC: GCAACAGCAAAGGGAAAGAAA | Tsingke Biotech | N/A |
| siNC: UUCUCCGAACGUGUCACGUTT | Tsingke Biotech | N/A |
| siKDM4A 1#: UUCGAGAGUUCCGCAAGAUAG | Tsingke Biotech | N/A |
| siKDM4A 2#: GCACCGAGUUUGUCUUGAAAU | Tsingke Biotech | N/A |
| siPHRF1 1#: CAGUUGAUCGAACUCUAUUUA | Tsingke Biotech | N/A |
| siPHRF1 2#: UUGAGAGCUUCCGGAUCAAUA | Tsingke Biotech | N/A |
| Plasmid | | |
| Cloning XPC to pLVX-IRES-Puro plasmid | Wang et al | N/A |
| PHRF1 to PCDNA3.1 plasmid (NM_001286581) | Youbio | Cat# 46112 |
| RFP-LC3B plasmid | Jiqin Lian, Prof. | N/A |
